# The Trait Repertoire Enabling Cyanobacteria to Bloom Assessed through Comparative Genomic Complexity and Metatranscriptomics

**DOI:** 10.1128/mBio.01155-20

**Published:** 2020-06-30

**Authors:** Huansheng Cao, Yohei Shimura, Morgan M. Steffen, Zhou Yang, Jingrang Lu, Allen Joel, Landon Jenkins, Masanobu Kawachi, Yanbin Yin, Ferran Garcia-Pichel

**Affiliations:** a Department of Biological Sciences, Northern Illinois University, DeKalb, Illinois, USA; b Biodesign Center for Fundamental and Applied Microbiomics, Arizona State University, Tempe, Arizona, USA; c National Institute for Environmental Studies, Tsukuba, Ibaraki, Japan; d Biology Department, James Madison University, Harrisonburg, Harrisonburg, Virginia, USA; e Jiangsu Key Laboratory for Biodiversity and Biotechnology, School of Biological Sciences, Nanjing Normal University, Nanjing, Jiangsu, China; f U.S. Environmental Protection Agency Office of Research and Development, Cincinnati, Ohio, USA; g Nebraska Food for Health Center, Department of Food Science and Technology, University of Nebraska-Lincoln, Lincoln, Nebraska, USA; Oregon State University

**Keywords:** cyanobacterial bloom, comparative genomics, ecophysiology, genomic complexity, metatranscriptome, *Microcystis aeruginosa*, adaptation, cyanobacteria, ecogenomics, water blooms

## Abstract

Water bloom development due to eutrophication constitutes a case of niche specialization among planktonic cyanobacteria, but the genomic repertoire allowing bloom formation in only some species has not been fully characterized. We posited that the habitat relevance of a trait begets its underlying genomic complexity, so that traits within the repertoire would be differentially more complex in species successfully thriving in that habitat than in close species that cannot. To test this for the case of bloom-forming cyanobacteria, we curated 17 potentially relevant query metabolic pathways and five core pathways selected according to existing ecophysiological literature. The available 113 genomes were split into those of blooming (45) or nonblooming (68) strains, and an index of genomic complexity for each strain’s version of each pathway was derived. We show that strain versions of all query pathways were significantly more complex in bloomers, with complexity in fact correlating positively with strain blooming incidence in 14 of those pathways. Five core pathways, relevant everywhere, showed no differential complexity or correlations. Gas vesicle, toxin and fatty acid synthesis, amino acid uptake, and C, N, and S acquisition systems were most strikingly relevant in the blooming repertoire. Further, we validated our findings using metagenomic gene expression analyses of blooming and nonblooming cyanobacteria in natural settings, where pathways in the repertoire were differentially overexpressed according to their relative complexity in bloomers, but not in nonbloomers. We expect that this approach may find applications to other habitats and organismal groups.

## Abstract (precis)

We pragmatically delineate the trait repertoire that enables organismal niche specialization. We based our approach on the tenet, derived from evolutionary and complex-system considerations, that genomic units that can significantly contribute to fitness in a certain habitat will be comparatively more complex in organisms specialized to that habitat than their genomic homologs found in organisms from other habitats. We tested this in cyanobacteria forming harmful water blooms, for which decades-long efforts in ecological physiology and genomics exist. Our results essentially confirm that genomics and ecology can be linked through comparative complexity analyses, providing a tool that should be of general applicability for any group of organisms and any habitat, and enabling the posing of grounded hypotheses regarding the ecogenomic basis for diversification.

## INTRODUCTION

Functional niche specialization is a ubiquitous trait in microorganisms, a fact that is reflected in their trait and genomic repertoires (1). The presence of a given trait results from positive selection by environmental challenges typical of a particular habitat. In contrast, selection will tend to be negative for traits that do not contribute much to the fitness in the habitat under consideration, since they will be costly to encode and express for no or minimal benefit (2). Two factors determine through selection if a trait will be a part of the specialized repertoire addressing a given environmental challenge: the fitness contribution it provides during challenge and the cost of encoding and expressing the genomic units on which it is based (3). For example, the ability to synthesize highly effective nitrogenous compatible solutes in the adaptation of cyanobacteria to hypersaline media only occurs in extremely halophilic forms (4), whereas moderately halotolerant species settle for the use of disaccharides. However, this dual black-and-white distinction is unlikely to be always patent in nature, because habitats are variable in space and time, and because traits can be of relevance in more than one environment. To follow on our example, the use and expression of disaccharide-compatible solutes also confer significant fitness to soil cyanobacteria (5) even though most soils become hyperosmotic only when severely dry.

Hence, genomic systems may provide variable fitness depending on the frequency and preponderance of an environmental challenge in different environments. Under this framework, it is also logical to hypothesize that pathways or genomic systems that provide specific adaptive solutions to an environmental challenge will be comparatively more complex or sophisticated in species that are often challenged than in those living in habitats where the system brings only marginal or occasional fitness. This is because the increased cost of system maintenance of a complex system will be easily offset by the fitness benefits of a fine-tuned, effectively functioning configuration—if it is used extensively. A high-maintenance, complex system may not be affordable when it is only necessary occasionally, such that organisms settle for less effective or nimble, but simpler, alternatives through adaptive gene loss, or even outsourcing parts of a pathway to cooccurring organisms (6). This hypothesis is consistent with theoretical considerations in systems biology regarding the robustness versus failure of complex evolvable systems (7), the distinction between explicit versus “underground” metabolism (8), and the tendency to add complexity to existing nodes during system growth in metabolic network theory (9). One prominent example is the cellulosome in specialized cellulolytic bacteria (e.g., Clostridium thermocellum), a multienzyme complex with as many as 160 enzymes (10), where each enzyme also has multiple paralog genes (11). Other organisms (yeast, for example) that can grow on cellulose albeit inefficiently, make do with an unorganized subset of these genes (12). Similarly, uptake systems become demonstrably more complex under nutrient limitation (13), as do antibiotic resistance genes in the continued presence of antibiotics (14). Such relationships between complexity and functional relevance should be principally quantifiable.

To test this, we hypothesized that planktonic cyanobacteria that form blooms have and express specific pathways useful with the constraints of life in a bloom, and that these pathways will be more complex than their cognates in other nonblooming forms. We chose this example to study the functional niche specialization of water bloom-forming cyanobacteria at the systems level, because their niche is well defined among the many occupied by cyanobacteria, and only some species are able to form these blooms, even among a much larger diversity of extant planktonic forms (15). Also, cyanobacterial blooms (often referred to as CyanoHABs, for cyano-harmful algal blooms) have been well studied at the ecological, physiological, and genomic levels, so there is a considerable database of information that can help determine correlations of the complexity of certain genomic traits with their known or suspected differential relevance for bloom formation. These blooms are primarily driven by elevated nutrient loading from human activities (16, 17), typically occurring within a short period of time and particularly in waters of long residence time (18), which leaves no time for within-bloom evolution of new functions given the low mutation rate of cyanobacteria (19). Their consistent dominance in eutrophic oceans and inland waters has now expanded to a global scale of increasingly eutrophic waters (20, 21), suggesting that their genomes must be preequipped with effective functional capabilities to take advantage of the elevated resources that were not available in the preeutrophication era.

Some of the important traits for bloom formers have been inferred either through field studies or through nutrient manipulation in laboratory settings. Functions held to be important include those related to resource utilization such as buoyancy generation encoded by a *gvp* gene cluster (22), utilization of macronutrients (carbon, nitrogen, phosphate, and sulfur), trace elements (17, 23), and small-molecule organics, such as amino acid and simple sugars (24). Stress-resistance pathways are sometimes deemed important, including biosynthesis of ultraviolet sunscreens (25), toxin production (16), heavy metals (26, 27), or antibiotic resistance (28). However, further evaluation is needed because, first, the general occurrence of these pathways in bloomers has not been systematically evaluated, but rather inferred from one or few species (17). Second, metabolomic context and contrast with nonblooming controls are typically lacking (23). Despite these challenges, establishing the consensus of physiology for bloom formation remains a priority for effective bloom control (29).

Here, we apply a comparative genomic perspective derived from our “significance begets complexity” hypothesis, to address this goal using correlational analyses. We categorized 113 cyanobacterial strain genomes according to their tendency to form blooms. We then reconstructed pathways of interest, characterized their complexity pragmatically in each genome using coding lengths and number of identifiable genetic components, and probed the relationships between the two parameters statistically. We then used meta-transcriptomics of natural cyanobacterial planktonic populations to validate our results.

## RESULTS

### Phylogeny, genomic features, and morphology across the data set.

**FIG 1:**
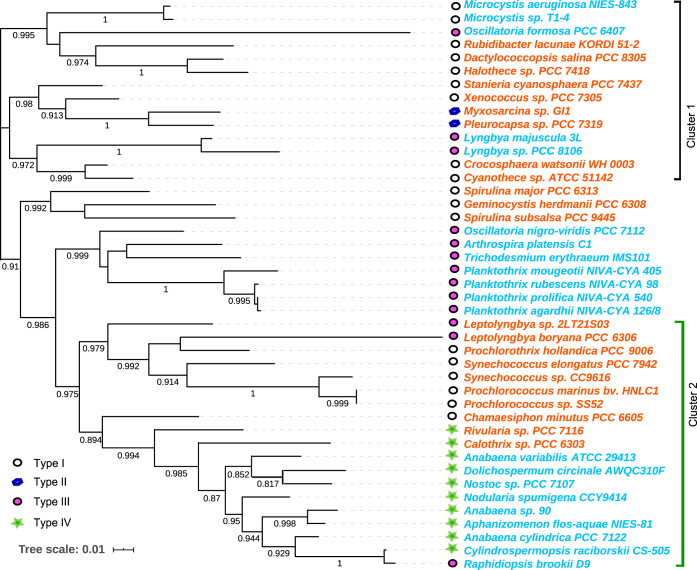
Phylogeny of the 43 cyanobacterial strains used based on 16S rRNA gene sequences. Blooming species are in blue and nonblooming in red. Morphological types are indicated according to the traditional Rippka groups.

The 113 genomes were initially classified as bloomers (45 genomes) and nonbloomers (68 genomes) (Table S1 in the supplemental material). As shown in Fig. 1 and Fig. S1, both blooming and nonblooming genomes appeared recurrently within the same phylogenetic cluster, such as clusters 1 and 2 of Fig. 1. With respect to morphology, the set of bloomers included unicellular, colonial, and filamentous cyanobacteria. Specifically, bloomers and nonbloomers from our set had representatives in groups I, III, and IV, although the two representatives of group II were both bloomers (Fig. 1). Besides similarities in phylogeny and morphology, the sets of bloomers and nonbloomers also had similar genomic features, such as genome size, gene content or GC content (two-sample Wilcoxon test, *P > *0.05), and there were no apparent correlations between their ability to form blooms (Blooming Incident Index, see below) and general genomic features, although bloomers had a narrower range (Fig. S2). All of this supports the notion that our comparative data set did not significantly bias according to phylogeny, morphology, or rough genomic traits.

10.1128/mBio.01155-20.1 FIG S1 Phylogeny of the 43 cyanobacterial strains used based on 16S rRNA sequences. Download FIG S1, 1.0 MB. Copyright © 2020 Cao et al. 2020 Cao et al. This content is distributed under the terms of the Creative Commons Attribution 4.0 International license.

10.1128/mBio.01155-20.2 FIG S2 The relationship between bloom incidence index (BII) and cell shape and genomic features in 113 cyanobacterial strains. Download FIG S2, PDF file, 0.2 MB. Copyright © 2020 Cao et al. 2020 Cao et al. This content is distributed under the terms of the Creative Commons Attribution 4.0 International license.

10.1128/mBio.01155-20.7 TABLE S1 The 113 strains used in this study. Download Table S1, DOC file, 0.4 MB. Copyright © 2020 Cao et al. 2020 Cao et al. This content is distributed under the terms of the Creative Commons Attribution 4.0 International license.

### Overview of the curated ecophysiological pathways in cyanobacteria.

**FIG 2:**
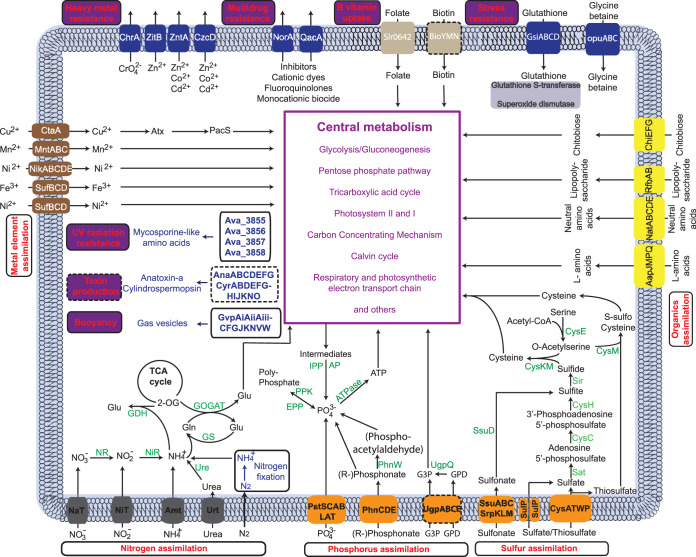
The core and query pathways in *Aphanizomenon flos-aquae* NIES-81. Each component is detailed in the corresponding tables (Tables S2 to S24). The pathways labeled with dashed borders are not complete (due to the absence of required components) in this strain but may be complete in others.

We reconstructed 5 core metabolic pathways and 17 query pathways derived from existing ecological tests in the literature (Table S2). Core pathways were used as controls because, if our hypothesis holds, they should not show differential complexity according to habitat. Core pathways included glycolysis, TCA cycle (TCA), pentose phosphate pathway (PPP), the electron transport chain (ETC), and the Calvin cycle (Calvin). These are typically of high similarity and low variability among microbial strains (30). Two modules of photosynthesis—PBS (phycobilisome) and CCM (CO_2_-concentrating mechanism)—were involved in utilization of external resources (light and CO_2_), and thus were formally included as query pathways in the comparison between bloomers and nonbloomers, even though one could also argue they are core pathways for phototrophs. The other query pathways selected have been shown to promote cyanobacterial growth and bloom formation (see references in Text S1 the supplemental material; Table S2). We illustrate the query pathways for the case of Aphanizomenon flos-aquae strain NIES-81 (Fig. 2). More detailed illustrations of all core and query pathways are provided in Fig. S3. *A. flos-aquae* NIES-81 has pathways for the assimilation of carbon (CCM), nitrogen (N), phosphorus (P), sulfur (S), free amino acids (AAT), and sugars, as well as trace elements and vitamins (TEVit). It has capabilities against stressors like heavy metals (MetalR), antibiotics (DrugR), oxidative stress (OSR), UV radiation (MAA), osmotic pressure, and possibly predation (through toxin production). Additionally, buoyancy can be regulated through the biosynthesis of gas vesicles. These pathways had different composition (number of components, e.g., transporters or enzymes) and complexity (components consisting of multiple subunits) among different strains.

10.1128/mBio.01155-20.9 TEXT S1 Supplemental methods and references. Download Text S1, DOCX file, 0.03 MB. Copyright © 2020 Cao et al. 2020 Cao et al. This is an open-access article distributed under the terms of the Creative Commons Attribution 4.0 International license.

10.1128/mBio.01155-20.3 FIG S3 Core and query pathway in this study. Download FIG S3, PDF file, 1.7 MB. Copyright © 2020 Cao et al. 2020 Cao et al. This content is distributed under the terms of the Creative Commons Attribution 4.0 International license.

10.1128/mBio.01155-20.8 TABLE S2 The classification of central and query pathways and their protein reference sources. Download Table S2, DOC file, 0.3 MB. Copyright © 2020 Cao et al. 2020 Cao et al. This content is distributed under the terms of the Creative Commons Attribution 4.0 International license.

### Complexity difference in pathways between bloomers and nonbloomers.

On average, bloomers had a total of 461 (±57) proteins in the 19 query pathways, accounting for about 14.5% of their entire genomes, whereas nonbloomers encoded only 274 (±42) proteins, accounting for 10.3% of the genes, suggesting there is indeed a tendency for more complex query pathways among bloomers. As a control, core pathways had on average similar number of proteins between bloomers and nonbloomers, 169 versus 160, accounting for 5.3% and 6% of the total genes, respectively. The specific components of the core and query pathways for each strain in our analyses can be found as Tables S3 to S16 at https://github.com/hshcao/CHABpathways.

**FIG 3:**
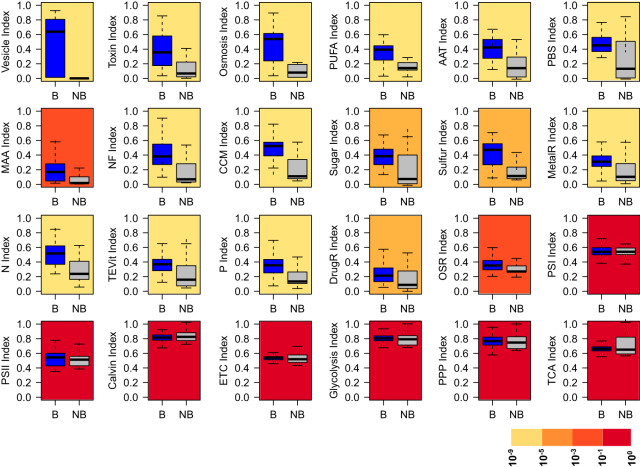
Average relative complexity (GCI) in blooming and nonblooming strains for each of the 24 pathways. B, blooming; NB, nonblooming. The background color of each plot represents the *P* values of the comparison between blooming and nonblooming strains, based on Wilcoxon sign-rank tests.

More specifically, we present the ratio between bloomers and nonbloomers of each of our eight genomic complexity metrics in Table 1. The metrics considered the number of components in each pathway, component complexity (e.g., multisubunits of enzyme complexes or ABC transporters), and the proportion of encoding sequence of the entire genome for these components (see the Materials and Methods). Again here, query pathways (except those related to photosynthesis) were significantly (Wilcoxon sign-rank test, *P* < 0.05) more complex among bloomers regardless of metric. However, the magnitude of the difference varied considerably with pathway, from almost 7-fold in some (vesicle) to marginal values in others (ORS). The ratios were quite consistent among metrics. As expected, the core metabolic pathways did not differ significantly between bloomers and nonbloomers for any complexity metric. PSII and PSI were only marginally different, however, perhaps because they should be considered core pathways among phototrophs. Table 1 also includes the ratios of a general genomic complexity index (GCI, computed as an average of the eight individual complexity metrics). Clearly, the vesicle pathway had the highest GCI ratio (6.6), followed by toxin, PUFA (polyunsaturated fatty acids), osmosis, AAT, PBS, MAA, nitrogen fixation (NF), and CCM. It is noteworthy that none of the nonbloomers had a complete set of genes for toxin production, so some of the metrics could not be calculated (with zeros as denominator), indicating that our comparison in this case is an underestimate. In Fig. 3 we show the actual distribution of GCI for bloomers and nonbloomers. According to this integrated comparison, none of the core pathways (including PSI and PSII) were different. Among the query pathways, all were significantly more complex among bloomers.

**TABLE 1:** Ratio of complexity metrics of core and query pathways between blooming and nonblooming strains

Pathway	Mean ratio	Ratio (*P* value)^*a*^^,^^*b*^							
		NP	NC	NPC	LP	FLP	LC	FLC	PC
Vesicle	6.6	**6.9 (2 × 10^−8^)**	**6.6 (2 × 10^−8^)**	**6.9 (2 × 10^−8^)**	**6.5 (2 × 10^−8^)**	**6.5 (4 × 10^−8^)**	**6.5 (2 × 10^−8^)**	**6.5 (4 × 10^−8^)**	**6.6 (2 × 10^−8^)**
Toxin	2.5	**2.0 (1 × 10^−7^)**	NA	NA	**3.0 (1 × 10^−8^)**	**2.5 (7 × 10^−7^)**	NA	NA	NA
Osmosis	2.4	**2.2 (3 × 10^−7^)**	**2.6 (6 × 10^−5^)**	**2.5 (7 × 10^−4^)**	**2.2 (2 × 10^−7^)**	**1.9 (3 × 10^−6^)**	**2.5 (8 × 10^−4^)**	**2.8 (1 × 10^−4^)**	**2.6 (6 × 10^−5^)**
PUFA	2	**2.1 (5 × 10^−8^)**	**1.4 (2 × 10^−7^)**	**1.6 (1 × 10^−6^)**	**3.4 (3 × 10^−8^)**	**3.0 (5 × 10^−7^)**	**2.0 (3 × 10^−6^)**	1.1 (1 × 10^−1^)	**1.4 (2 × 10^−7^)**
AAT	1.9	**1.7 (2 × 10^−8^)**	**2.2 (7 × 10^−8^)**	**2.2 (3 × 10^−8^)**	**1.8 (7 × 10^−9^)**	**1.3 (2 × 10^−6^)**	**2.1 (5 × 10^−6^)**	**1.8 (9 × 10^−3^)**	**2.1 (7 × 10^−8^)**
PBS	1.8	**1.7 (1 × 10^−5^)**	**1.7 (4 × 10^−5^)**	**1.7 (1 × 10^−5^)**	**1.8 (2 × 10^−6^)**	**1.6 (4 × 10^−5^)**	**2.0 (8 × 10^−7^)**	**2.0 (8 × 10^−8^)**	**1.6 (4 × 10^−5^)**
MAA	1.7	**1.5 (2 × 10^−4^)**	1.7 (2 × 10^−1^)	1.6 (2 × 10^−1^)	**2.0 (1 × 10^−5^)**	1.7 (5 × 10^−2^)	**1.5 (2 × 10^−2^)**	**1.7 (3 × 10^−2^)**	1.7 (2 × 10^−1^)
NF	1.7	**1.7 (6 × 10^−7^)**	**1.5 (1 × 10^−5^)**	**1.8 (6 × 10^−7^)**	**1.9 (6 × 10^−8^)**	**1.5 (8 × 10^−7^)**	**2.0 (6 × 10^−8^)**	**1.7 (4 × 10^−8^)**	**1.5 (1 × 10^−5^)**
CCM	1.7	**1.9 (2 × 10^−10^)**	**1.5 (3 × 10^−10^)**	**2.2 (7 × 10^−11^)**	**1.8 (4 × 10^−10^)**	**1.3 (8 × 10^−7^)**	**2.0 (6 × 10^−11^)**	**1.4 (1 × 10^−6^)**	**1.5 (3 × 10^−10^)**
Sugar	1.6	**1.7 (8 × 10^−5^)**	**1.4 (5 × 10^−3^)**	**1.6 (1 × 10^−4^)**	**1.8 (5 × 10^−5^)**	**1.6 (7 × 10^−4^)**	**1.8 (3 × 10^−5^)**	**1.6 (1 × 10^−3^)**	**1.4 (5 × 10^−3^)**
Sulfur	1.6	**1.9 (2 × 10^−10^)**	**1.3 (5 × 10^−10^)**	**2.1 (2 × 10^−11^)**	**1.9 (2 × 10^−10^)**	**1.2 (2 × 10^−5^)**	**2.0 (3 × 10^−11^)**	**1.3 (5 × 10^−5^)**	**1.3 (5 × 10^−10^)**
MetalR	1.5	**1.5 (7 × 10^−7^)**	**1.6 (8 × 10^−8^)**	**1.6 (2 × 10^−7^)**	**1.6 (1 × 10^−6^)**	**1.3 (5 × 10^−5^)**	**1.5 (9 × 10^−7^)**	**1.1 (2 × 10^−2^)**	**1.6 (8 × 10^−8^)**
N	1.4	**1.6 (3 × 10^−9^)**	**1.3 (2 × 10^−8^)**	**1.6 (2 × 10^−8^)**	**1.7 (7 × 10^−10^)**	1.1 (8 × 10^−2^)	**1.7 (5 × 10^−9^)**	1.1 (4 × 10^−1^)	**1.3 (2 × 10^−8^)**
TEVit	1.4	**1.4 (6 × 10^−6^)**	**1.4 (2 × 10^−6^)**	**1.5 (1 × 10^−5^)**	**1.5 (9 × 10^−7^)**	1.1 (4 × 10^−1^)	**1.4 (2 × 10^−5^)**	**1.0 (1 × 10^−5^)**	**1.4 (2 × 10^−6^)**
P	1.3	**1.5 (2 × 10^−6^)**	**1.2 (2 × 10^−4^)**	**1.4 (9 × 10^−5^)**	**1.6 (9 × 10^−7^)**	1.1 (1 × 10^−1^)	**1.6 (1 × 10^−5^)**	1.1 (4 × 10^−1^)	**1.2 (2 × 10^−4^)**
DrugR	1.3	**1.5 (1 × 10^−5^)**	**1.5 (1 × 10^−3^)**	**1.3 (8 × 10^−4^)**	**1.6 (1 × 10^−5^)**	0.6 (3 × 10^−1^)	**1.1 (8 × 10^−3^)**	1.2 (2 × 10^−1^)	**1.5 (1 × 10^−3^)**
ORS	1.1	**1.3 (2 × 10^−6^)**	1.0 (4 × 10^−1^)	**1.3 (3 × 10^−6^)**	**1.3 (5 × 10^−7^)**	**0.8 (2 × 10^−4^)**	**1.4 (9 × 10^−7^)**	**0.9 (2 × 10^−4^)**	1.0 (4 × 10^−1^)
PSI	1	**1.1 (5 × 10^−7^)**	**1.2 (5 × 10^−8^)**	**1.1 (2 × 10^−6^)**	**1.0 (2 × 10^−3^)**	**0.6 (2 × 10^−12^)**	**1.0 (3 × 10^−3^)**	**0.6 (2 × 10^−12^)**	**1.2 (2 × 10^−9^)**
PSII	1	**1.1 (6 × 10^−4^)**	**1.2 (3 × 10^−5^)**	**1.1 (7 × 10^−4^)**	1.0 (4 × 10^−1^)	**0.6 (1 × 10^−7^)**	1.0 (4 × 10^−1^)	**0.6 (2 × 10^−7^)**	**1.2 (3 × 10^−5^)**
Calvin	0.9	1.0 (1 × 10^−1^)	1.0 (3 × 10^−1^)	1.0 (7 × 10^−1^)	1.0 (4 × 10^−1^)	0.7 (6 × 10^−2^)	1.0 (4 × 10^−1^)	0.6 (5 × 10^−2^)	1.0 (3 × 10^−1^)
Glycolysis	0.9	1.0 (9 × 10^−1^)	1.0 (7 × 10^−1^)	1.0 (7 × 10^−1^)	1.0 (5 × 10^−1^)	0.7 (8 × 10^−2^)	1.0 (5 × 10^−1^)	0.9 (5 × 10^−2^)	1.0 (7 × 10^−1^)
ETC	0.9	1.0 (1 × 10^−1^)	1.0 (2 × 10^−1^)	1.0 (8 × 10^−1^)	1.0 (6 × 10^−1^)	0.8 (9 × 10^−2^)	1.0 (8 × 10^−1^)	0.8 (8 × 10^−2^)	1.0 (2 × 10^−1^)
PPP	1.0	1.0 (5 × 10^−1^)	1.0 (3 × 10^−1^)	1.0 (7 × 10^−1^)	1.0 (4 × 10^−1^)	0.8 (6 × 10^−2^)	1.0 (7 × 10^−1^)	0.9 (5 × 10^−2^)	1.0 (6 × 10^−1^)
TCA	0.9	1.0 (5 × 10^−1^)	1.0 (5 × 10^−1^)	1.0 (7 × 10^−1^)	1.0 (5 × 10^−1^)	0.9 (5 × 10^−2^)	1.0 (3 × 10^−1^)	0.7 (5 × 10^−2^)	1.0 (7 × 10^−1^)

a NP, total number of proteins in pathway; NC, total number of protein complexes (including multiprotein complexes and singular proteins); NPC, total number of proteins in the complete multiprotein complexes; LP, total length (in base pairs) of nucleotide sequences encoding all the proteins in pathway; FLP, fraction of total coding length of proteins (the ratio of LP to genome size); LC, total length (in base pairs) of proteins in the complete multiprotein complexes; FLC, fraction of total coding length of proteins in the complete multiprotein complexes (the ratio of LC to genome size); PC, the ratio of NC to the total number of complexes in the reference protein set; NA, not applicable.

b *P* values indicate the significance of the difference between numerator and denominator of each ratio according to Wilcoxon sign-rank tests. Significant ratios are in boldface.

Because some species were overrepresented with multiple strain genomes in our data set, such as Microcystis aeruginosa, Planktothrix agardhii, *Cyanothece* sp., *Synechococcus* sp., Crocosphaera watsonii, Prochlorococcus marinus and *Prochlorococcus* sp., we carried out additionally analyses with data sets that used only one random genome for each species. A similar pattern in complexity differences was still observed (Fig. S4).

10.1128/mBio.01155-20.4 FIG S4 Comparison of the 24 pathways in terms of eight metrics between blooming and nonblooming strains without overrepresented species. Download FIG S4, PDF file, 0.2 MB. Copyright © 2020 Cao et al. 2020 Cao et al. This content is distributed under the terms of the Creative Commons Attribution 4.0 International license.

The pattern shown in Fig. 3 may potentially be artifactual due to the presence of evolutionary/phylogenetic legacies, given that the set of strains was not randomly distributed in the cyanobacteria phylogeny, but rather tended to aggregate in certain clades. To test for such an effect, we selected two clusters characterized by different morphology and blooming types (clusters 1 and 2 in Fig. 1) and performed the comparison between blooming and nonblooming species within each. In these analyses, blooming species still had higher metrics (Wilcoxon sign-rank test, all *P* values < 10^−2^) even when determined among closely related phylogenetic strains. We conclude that any effects of evolutionary/phylogenetic legacies were insignificant.

**FIG 4:**
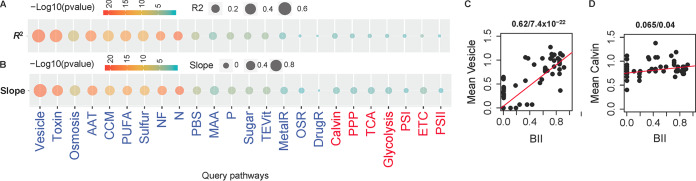
Bubble plots of Pearson’s correlations between GCI and BII in each pathway. The circle size represents either the coefficient of determination (*R*^2^) (A) or the slope of the correlation (B) with the significance (*P*) of the correlation indicated with colors. Two correlations are also shown as examples: Vesicle (C) and Calvin cycle (D).

We then probed in a more quantitative way the relevance of the query pathways to bloom formation by correlation, because some of the strains are not always exclusively bloomers or nonbloomers and may be referenced in the literature under either class. For each pathway we ran linear correlations between the GCI and a bloom incidence index (BII; derived from the literature, see the Materials and Methods). Expectedly, we found no significant correlation (*P* > 0.05) for any of the core pathways or PSI and PSII (Fig. 4; see detailed plot in Fig. S5). Among the query pathways, correlations were significant for all pathways except for OSR, DrugR, and MetalR. However, only in 7 pathways (vesicle, toxin, PUFA and AAT, CCM, sulfur, and osmosis) were the correlation coefficients large (*R*^2^ >0.4), and only 2 additional pathways had *R*^2^ >0.25 (NF and N). Interestingly, besides the significance of the correlations, the slopes of the correlation also showed a similar pattern among the query pathways (Fig. 4B). Vesicle and Calvin were shown as examples of significant correlation (Fig. 4C) and of no correlation (Fig. 4D), respectively. We also calculated the correlation between BII and PCI of all genomes, i.e., a test of the effect of genome redundancy, and a similar result was found to that without redundancy (Fig. S6).

10.1128/mBio.01155-20.5 FIG S5 Pearson’s correlations between GCI and BII in each pathway. Download FIG S5, PDF file, 2.0 MB. Copyright © 2020 Cao et al. 2020 Cao et al. This content is distributed under the terms of the Creative Commons Attribution 4.0 International license.

10.1128/mBio.01155-20.6 FIG S6 Correlation between the query pathway PCI and BII of all 113 genomes. Download FIG S6, PDF file, 0.5 MB. Copyright © 2020 Cao et al. 2020 Cao et al. This content is distributed under the terms of the Creative Commons Attribution 4.0 International license.

**FIG 5:**
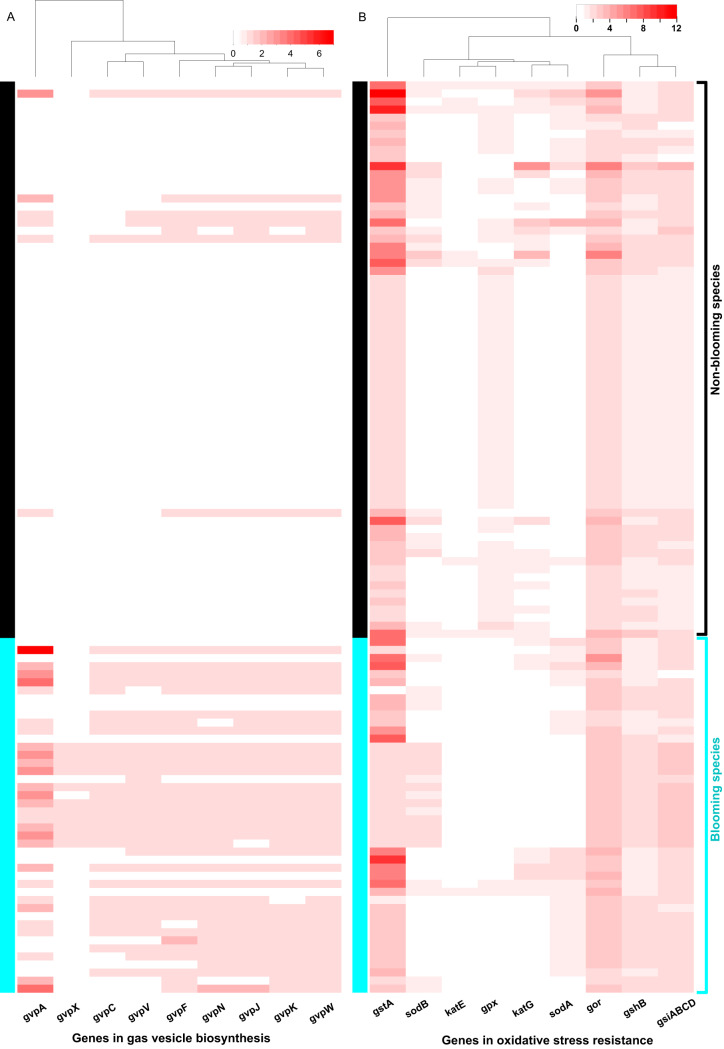
Gas vesicle (Vesicle) (A) and oxidative stress resistance (OSR) (B) genes present in the 113 genomes studied, sorted by blooming capacity.

To illustrate in detail how the query pathways identified above were indeed enriched with more components, we visualized the complete set of components in the significant vesicle pathway as opposed to the nonsignificant OSR, as shown in Fig. 5. For the vesicle pathway genes, all genes were more enriched in bloomers than in nonbloomers (Wilcoxon sign-rank test, *P* < 0.05). For the OSR genes, only the gene *gpx* was significantly higher in nonbloomers than bloomers (Wilcoxon sign-rank test, all *P* values < 10^−2^), while others were not different (Wilcoxon sign-rank test, *P* > 0.05).

### Correlation between GCI and the expression level of the query pathways in eutrophic and oligotrophic waters.

**FIG 6:**
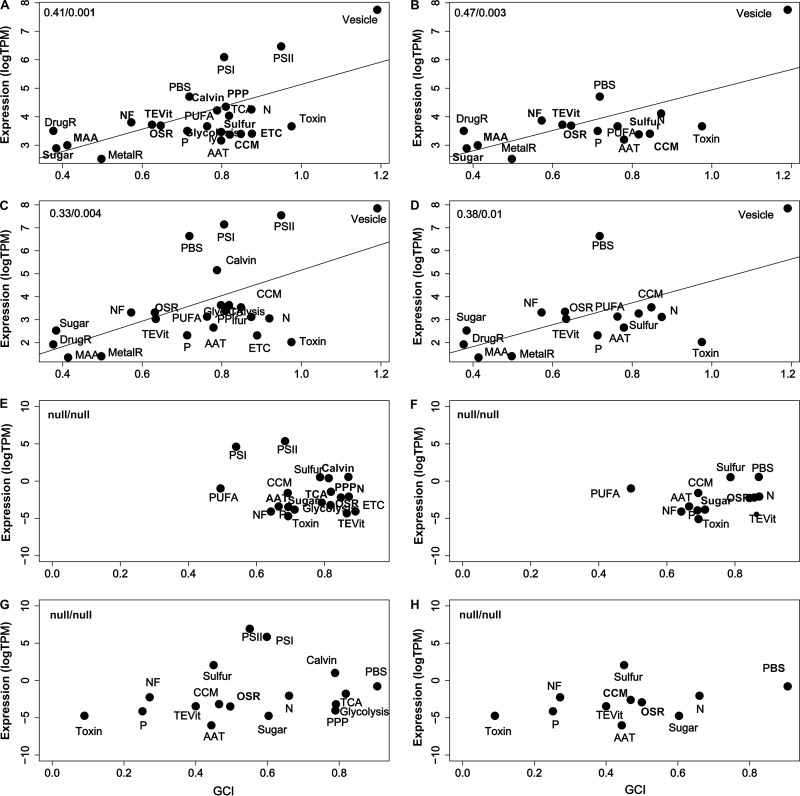
Correlations between the expression levels and GCI of the pathways studied in three different aquatic habitats. Metatranscriptomes were from eutrophic freshwater (Lake Erie, A and B; Harsha Lake, C and D), oligotrophic freshwater (Sparkling Lake, E and F), oligotrophic ocean water (western Arctic Ocean, Canada, G and H). Reference genomes representing the dominant species in these habitats were used for mapping: *M. aeruginosa* NIES-843 (Lake Erie and Harsha Lake), *Oscillatoria nigro-viridis* PCC7112 (Sparkling Lake), and *Synechococcus* sp. WH8102 (western Arctic Ocean, Canada). Pearson correlations were performed with (A, C, E, and G) or without (B, D, F, and H) core pathways. The coefficient of determination (*R*^2^) and the significance (*P*) of the correlations are on top left corner of each panel. GCI values were subject to angular transformation.

Finally, to test if the bloom-enabling genetic repertoire determined above was indeed relevant during bloom formation, we tested the prediction that the encoded genetic elements or pathways in the repertoire must be used in proportion to their relevance (and hence complexity). To that end, ecologically relevant metatranscriptomes of our own from eutrophic freshwater blooms in Lake Erie and Harsha Lake were studied in contrast to those of oligotrophic freshwater and oligotrophic ocean plankton which were publicly available. RNAseq reads were mapped to the genomes of the species closest to those dominating each habitat (*M. aeruginosa* strain NIES-843 for the bloom, Oscillatoria nigro-viridis strain PCC7112 for the oligotrophic freshwater plankton, and *Synechococcus* sp. strain WH8102 for the oligotrophic marine plankton). Significant correlations between the expression levels of pathways and the pathway GCIs were only found in blooming species in two eutrophic lakes (*R*^2^ = 0.47 and 0.38; both *P* < 0.01) (Fig. 6A to D), but not in nonbloomers in oligotrophic waters (Fig. 6E to H). Particularly, vesicle, PBS, CCM, and N were among those pathways highly expressed in blooming species, while nonsignificant pathways, such OSR, metalR, and even MAA, were expressed at the lowest levels. Interestingly, core pathways were expressed at moderate levels relative to the entire range of query pathway levels.

## DISCUSSION

A key understudied question in CyanoHAB research is whether there exists a common ecophysiology underlying the broad array of blooming species. We attempted to address this question from the comparative functional genomics perspective, testing the hypothesis that functional niche specialization will result in preferential sophistication of genomic systems that are differentially important for bloom formers. Such differential importance must be manifest in both encoding and expressing these functions. An initial comparison of pathway complexity among bloomers and nonbloomers found that pathway complexity (i.e., more coding investment) was statistically higher among bloomers for most potentially relevant pathways queried (and not for core pathways or core photosynthetic pathways), but also that the effect was not commensurate among pathways. The fact that none of the core metabolism was significantly different agrees with the general pattern of cyanobacterial genome evolution, i.e., the core set of genes is well conserved while accessory pathways are variable (30). The potential relevance of pathways for bloomers was mirrored in the level of expression of the query pathways in eutrophic waters but not in nonblooming species in oligotrophic waters. These results set the stage for direct experimental interrogation of the specific roles and mechanisms of each pathway.

### Pathways most enriched among blooming cyanobacteria.

We identified vesicle formation for buoyancy as the most enriched and highly expressed pathway, consistent with the ecological literature. Vesicle formation has been well established as part of the common ecophysiological repertoire among various types of bloomers, conferring buoyancy to adjust their position in the water column for optimal resource utilization (17, 23, 31). The toxin production pathway was also strongly enriched. While the exact role or benefit that bloomers derive from toxin production is still a matter of debate (32, 33), our data confirm their differential relevance for CyanoHABs. Their low expression may verify their potential role as a cell-cell signal molecule which is not required in large quantities (34). The relevance of these vesicle and toxin pathways found ample confirmation in how the complexity of these pathways best correlated with BII, and their level of expression in nature was commensurate with its high GCI.

Two pathways that saw significant enrichment among bloomers and a high correlation with BII, were the amino acid uptake systems, and the synthesis of polyunsaturated fatty acids. Cyanobacteria have been known to utilize organic nutrients such as glucose, acetyl-glucosamine, and various amino acids (24, 35), but the ecological roles of these organic nutrients have been little studied in bloom formation. As the level of organic matter, particularly free amino acids, increases in eutrophic waters (36–38), this pathway apparently becomes more important in driving or sustaining blooms, particularly in those cyanobacteria capable of mixotrophy (39). Recovering leaked amino acids from the exometabolome (40) becomes more feasible when cyanobacterial populations attain large density, as they do in blooms, since diffusional losses are counteracted by the high concentration of sources, unlike what would happen with dilute planktonic populations. The significance of PUFA, typically related to an ability to modify membrane fluidity in adaptation to temperature (41, 42), was surprising. Clearly temperature and its fluctuations are an important factor in lakes, but it is not clear to us how they are differentially more relevant than in other habitats. One possibility is that unsaturated fatty acids may help protect photosynthetic machinery in the presence of salt and other stresses (43, 44). It seems that increased attention should be paid to this issue in future research. The prominence of osmosis among the most responsive pathways was also rather surprising, given than salinity fluctuations are not among the most prominent traits in lakes, although they may be important for coastal or estuarine blooms.

### Role of macronutrients.

The role of macronutrient (C, N, P, or S) acquisition for bloom formation also found confirmation in our study. Particularly for C, N, and S, all correlated rather well with BII and were expressed at levels commensurate with their GCIs. For C, a diffusion limitation effect associated with high population density may enhance the value of sustaining complex carbon concentrating mechanisms (CCM) among bloomers, because heavy drawdown of CO_2_ can easily result in C limitation. In agreement with our results, rising CO_2_ level has been found to intensify phytoplankton blooms in eutrophic waters (45), and transporters and regulators of CCM seem very responsive to the changes in dissolved CO_2_ levels during a dense CyanoHAB (46). Interestingly, the correlation of P with BII was weak and its complexity differences between bloomers and nonbloomers were low (while both significant). According to our results, N and S acquisition would much more strongly define the bloom niche. Strikingly, field meta-analyses of over 1,000 lakes identified total nitrogen (TN), not total phosphorus, as the top nutrient for bloom formation (47). One possible reason for this is that bloom-forming and N-fixing cyanobacteria have a higher N requirement than other cyanobacteria due to their need for protein-based antenna pigments (phycobilisomes), and thus a higher N:P ratio (48) than our set of nonbloomers, in which some strains lack them (*Prochlorococcus* sp. and *Prochlorothrix*). But an *ad hoc* comparison excluding the latter did not support this notion, in that the significance remained below 0.01. In any event, this is not to say that P is not important. The even distribution of genes and particularly the fact that some blooming species have two sets of phosphate ABC transporters (*pstABCD*) suggests that P may simply not be more significant in blooms that in other habitats. In agreement with this view, external P additions do not necessarily lead to higher *Microcystis* abundance in Lake Erie where phosphorus level is already high (49), and the expression levels of P are similar to core pathways in all three habitats tested.

### Pathways of minor differential relevance.

Drug and metal resistance pathways had low correlations with BII and only minor increases in complexity among bloomers, and also showed lowest expression among the query pathways.

### The case of MAA sunscreens.

With respect to the water-soluble mycosporine pathway, which is common among cyanobacteria of many origins (50), we would like to note that increasing pathway length in this system is associated with the synthesis of mycosporines absorbing at longer wavelengths (from the UVB into the UVA) (51). It is possible that this fact may have contributed spuriously to enhance complexity in bloomers to reach a significance with no environmental meaning, because UVB radiation does not penetrate much in natural waters. This would tend to favor preferentially the use of (genomically complex) bi-substituted iminomycosporines among bloomers. Hence, the significance of MAA differences in complexity, and its correlation with BII, should be taken with caution despite that they were not among the most prominent differences.

### A tool of general applicability?

More generally, the data presented here provide support for the “relevance begets complexity” in comparative genomics. It delineates a tool to bring together comparative genomics and ecology. While this approach was here applied to the case of bloom-forming cyanobacteria, there is in principle no reason why this approach may not find application in a variety of other microbial habitats and organismal groups. On a broader scale, our approach can be combined with metagenome-assembled genomes to infer the link between genomic complexity and ecological niche, where together they could provide a comprehensive conceptual framework for predictive ecology from genomic data (52).

## MATERIALS AND METHODS

### Cyanobacterial genome sequences and strain information.

Whole-genome sequences of 113 cyanobacteria (Table S1) were downloaded from NCBI in May 2016. These cyanobacteria belong to 45 species. We initially classified them into blooming and nonblooming species based on a curated classification (16, 17, 53). The species not listed in the original curation were classified by querying their species/genus names in the Web of Science; if publications reporting blooms formation were found, they were classified as blooming and otherwise as nonblooming (Table S1).

A more precise ranking followed, in which we examined the blooming incidence index (BII) of all strains in the Web of Science. We used as query either the strain denomination (first), species epithet (if the strain denomination yielded insufficient hits), or, in a few cases, the genus name. The records returned were then further separated into genomic/physiological or environmental studies using appropriate key words. Finally, the records were classified into those that reported a bloom and those that did not. The BII for each strain was simply the proportion of environmental citations that mentioned blooming capacity, and it varied between 0 and 1 (Table S1). We discarded strains in genera that had <2 environmental citations. For convenience, and to check for phylogeny-derived patterns that may confound our search, we also classified the strains into four morpho-typical groups, according to the traditional divisions (54), as well as phylogenetically on the basis of 16S rRNA gene sequences (see details in the Methods in the supplemental material).

### Reference proteins of central and query pathways.

For core bacterial metabolic pathways, we included glycolysis, the pentose phosphate pathway, the TCA cycle, the Calvin cycle, and the electron transport chain. Additionally, we included four photosynthetic modules: photosystems I and II, phycobilisome, and CO_2_-concentrating mechanism. The reference protein sequences for the central metabolism were extracted from the SEED subsystems (55) and the KEGG database (56). The rest of the pathways (query pathways), which were presumably involved in differential resource utilization and stress resistance among bloomers, were manually curated from the literature and databases such as the SEED subsystems, KEGG, and the transporter database TCDB (57) (see the pathways in Table S2 and relevant citations in the Methods in the supplemental material).

### Homolog search in the cyanobacterial genomes.

With the reference proteins as the query, we searched against the 113 cyanobacterial genomes using BLASTP (58). We performed the search first with cyanobacteria-derived protein sequences if available; if not, heterotrophic bacteria-derived proteins were used. To ensure significant hits from BLASTP output, we used different E value and coverage thresholds for central pathways (E value of <10^−12^ and length coverage of >80%) and query pathways (E value of <10^−7^ and length coverage of >65%), since proteins in central pathways are more conserved than those in the query pathways (30). Among the 113 genomes, *Aphanizomenon flos-aquae* NIES-81 (59) was selected to illustrate central and query pathways.

### Assessing relevance of query pathways to CyanoHAB formation.

To quantify the comparative differences in complexity of any given pathway, we devised eight metrics that considered both the pathway composition and completeness (Table 1). The eight were as follows: NP (total number of proteins in each pathway and strain), NC (total number of protein complexes (including multiprotein complexes and single proteins per pathway and strain), NPC (total number of proteins in the complete multiprotein complexes per pathway and strain), LP (total base-pair length of nucleotide sequences encoding all the proteins in a pathway per strain), FLP (fraction of total coding length of proteins or the ratio of LP to strain’s genome size), LC (total base-pair length of proteins in the complete multiprotein complexes per strain and pathway), FLC (fraction of total coding length of proteins in the complete multiprotein complexes or the ratio of LC to the strain’s genome size), and PC (the ratio of NC to the total number of complexes in the reference protein set in each pathway per strain). Each metric was normalized so the lowest value found was set to 0 and the maximal value found in the set was set to 1. Once normalized, the 8 metrics of each strain and pathway were averaged to attain an overall genomic complexity index (GCI) for a pathway and strain that varied between 0 and 1. For statistical treatments, since GCI and BII are proportions, they were all subject to angular transformation.

### Correlation between GCI and the expression level of the query pathways.

We tested the correlation with metatranscrioptome of cyanobacteria collected from three types of water: (i) our own metagenomes from a CyanoHAB in eutrophic Lake Erie; (ii) oligotrophic freshwater Sparkling Lake; and (iii) the oligotrophic Arctic Ocean. The reads from each water body were mapped to reference genomes closest to their own dominant cyanobacterial species, *M. aeruginosa* NIES-843, *Oscillatoria nigro-viridis* PCC7112, and *Synechococcus* sp WH8102, respectively (see details in the Methods in the supplementary material). Next, the mean expression levels of each pathway were calculated as the average level of all the genes in the pathways. For each water body, the correlations between mean pathway (log transformed) expression levels and (angular transformed) BIIs were carried out for all (query and core) or only query pathways, using R.
