# Supplementary material for: The Trait Repertoire Enabling Cyanobacteria to Bloom Assessed through Comparative Genomic Complexity and Metatranscriptomics
Source: mBio. 2020 Jun 30;11(3):e01155-20. doi: 10.1128/mBio.01155-20 (PMC7327172; doi:10.1128/mBio.01155-20)
Supplement: FIG S1 [file mBio.01155-20-sf001.pdf]

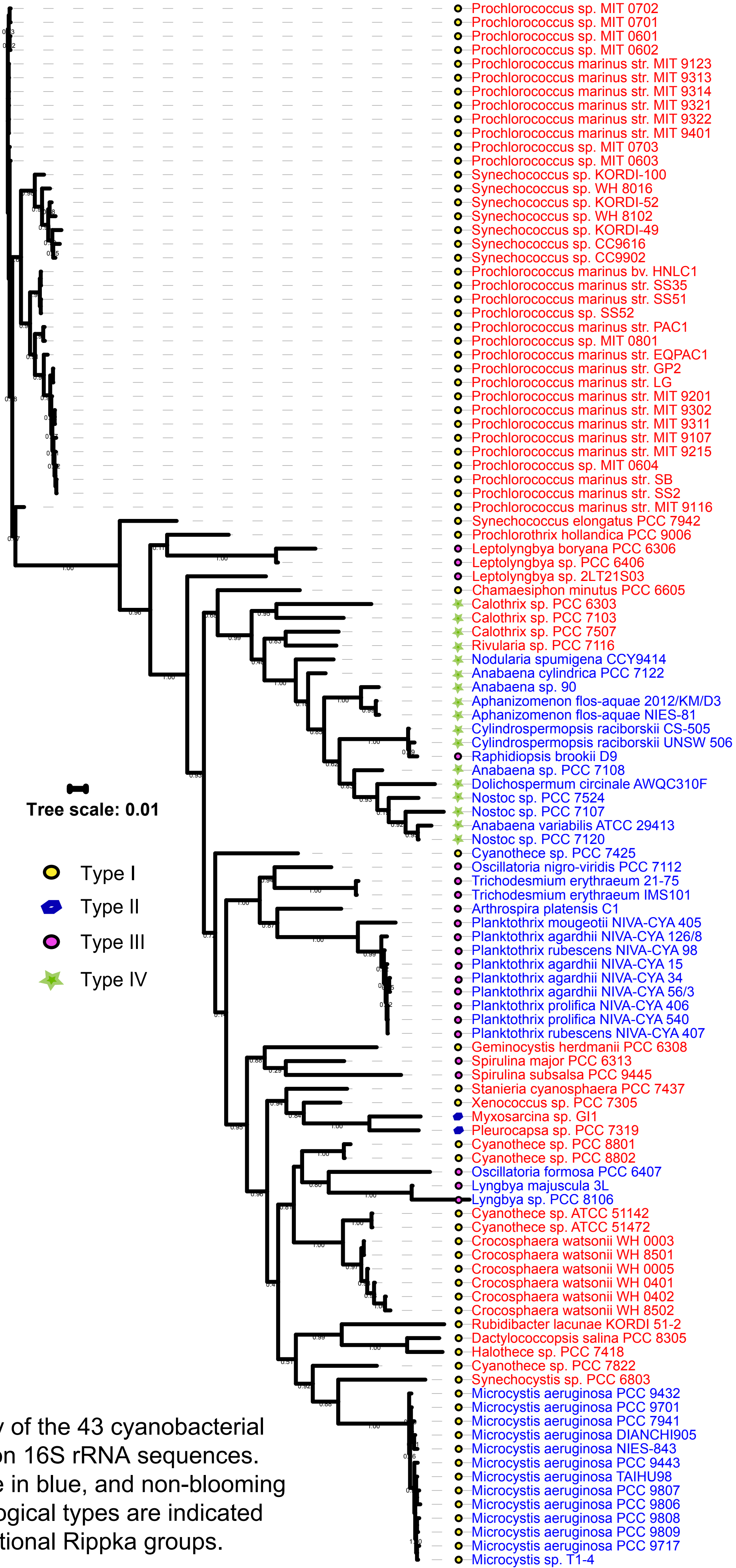

Figure S1. Phylogeny of the 43 cyanobacterial strains used based on 16S rRNA sequences. Blooming species are in blue, and non-blooming in red color. Morphological types are indicated according to the traditional Rippka groups.
