# Supplementary material for: The Trait Repertoire Enabling Cyanobacteria to Bloom Assessed through Comparative Genomic Complexity and Metatranscriptomics
Source: mBio. 2020 Jun 30;11(3):e01155-20. doi: 10.1128/mBio.01155-20 (PMC7327172; doi:10.1128/mBio.01155-20)
Supplement: FIG S2 [file mBio.01155-20-sf002.pdf]

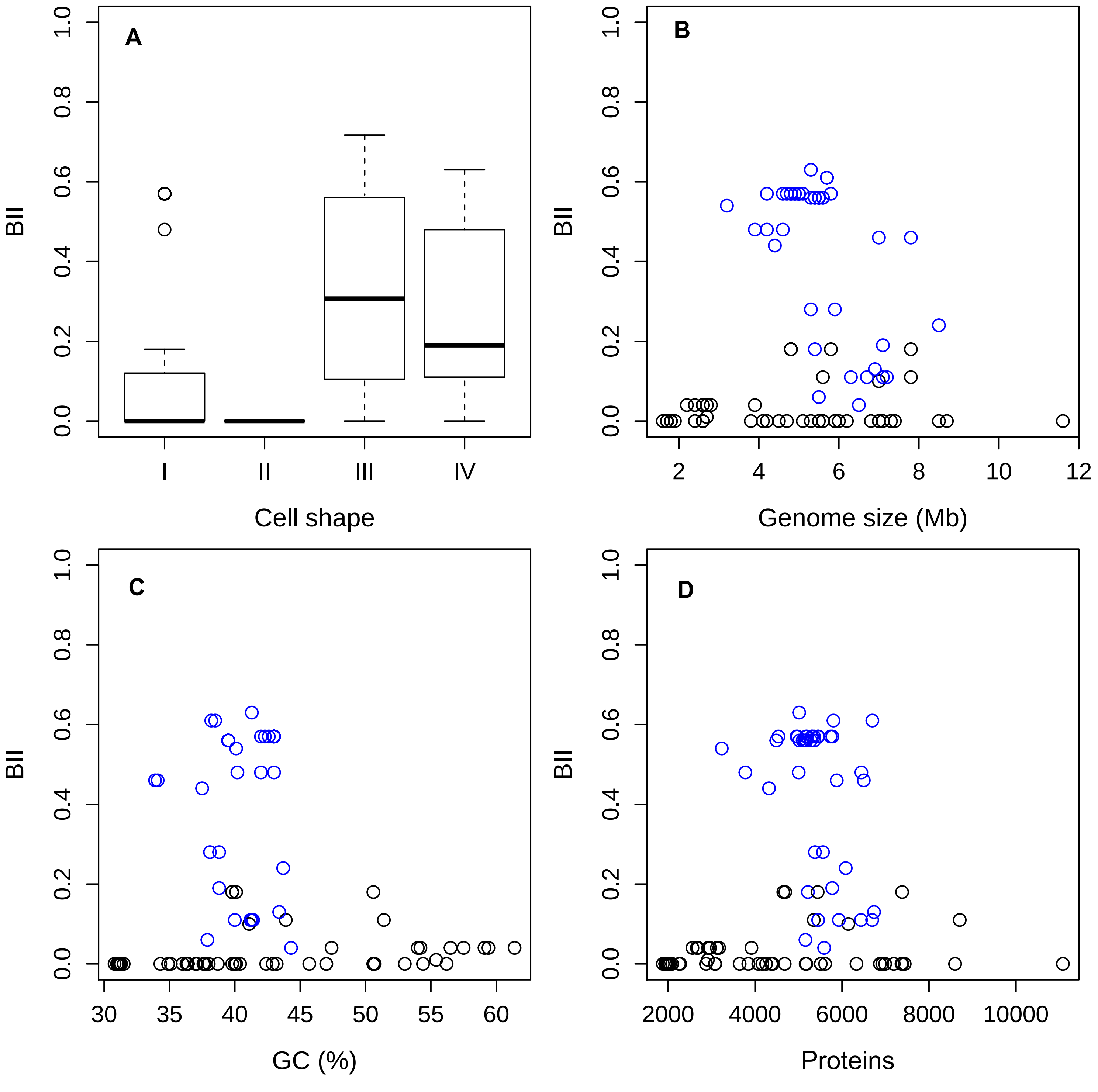

Figure S2. The relationship between bloom incidence index (BII) and cell shape and genomic features in 113 cyanobacterial strains. Colors of open circles indicate the blooming (blue) or non-blooming (black).
