# Supplementary material for: The Trait Repertoire Enabling Cyanobacteria to Bloom Assessed through Comparative Genomic Complexity and Metatranscriptomics
Source: mBio. 2020 Jun 30;11(3):e01155-20. doi: 10.1128/mBio.01155-20 (PMC7327172; doi:10.1128/mBio.01155-20)
Supplement: TABLE S1 [file mBio.01155-20-st001.doc]

**Table S1**. The 113 strains used in this study. The shape types are explained in the Methods. Blooming category is based on the specific strain source when it was collected. BI and BII is based on search in Web of Science using the species name and in some case genus name with sp. (e.g., BI: blooming incidence; BII: blooming incidence index. The blooming category was determined for each strain used, but the BII was calculated on the species name or genus name, which could have many other strains in the Web of Science.

| Strain | Genome size (Mb) | GC | CDS | Total Reports | Total Environmental Reports | BI | BII | Blooming category | Shape | Habitat |
| --- | --- | --- | --- | --- | --- | --- | --- | --- | --- | --- |
| Anabaena_cylindrica_PCC_7122 | 7.1 | 38.8 | 5775 | 732 | 112 | 21 | 0.19 | B | IV | freshwater |
| Anabaena_sp._90 | 5.3 | 38.1 | 5377 | 2419 | 577 | 160 | 0.28 | B | IV | freshwater |
| Anabaena_sp._PCC_7108 | 5.9 | 38.8 | 5562 | 2419 | 577 | 160 | 0.28 | B | IV | marine |
| Anabaena_variabilis_ATCC_29413 | 7.1 | 41.4 | 6436 | 1342 | 132 | 14 | 0.11 | B | IV | freshwater |
| Aphanizomenon_flos-aquae_2012/KM/D3 | 5.7 | 38.2 | 6698 | 571 | 446 | 271 | 0.61 | B | IV | freshwater |
| Aphanizomenon_flos-aquae_NIES-81 | 5.7 | 38.5 | 5803 | 571 | 446 | 271 | 0.61 | B | IV | freshwater |
| Arthrospira_platensis_C1 | 6.5 | 44.3 | 5590 | 815 | 251 | 10 | 0.04 | B | III | alkaline |
| Calothrix_sp._PCC_6303 | 7.0 | 39.8 | 6332 | 165 | 46 | 0 | 0 | N | IV | freshwater |
| Calothrix_sp._PCC_7103 | 11.6 | 40.4 | 11079 | 165 | 46 | 0 | 0 | N | IV | freshwater |
| Calothrix_sp._PCC_7507 | 7.0 | 43.2 | 6927 | 165 | 46 | 0 | 0 | N | IV | freshwater |
| Chamaesiphon_minutus_PCC_6605 | 6.8 | 45.7 | 7368 | 2 | 1 | 0 | 0 | N | I | freshwater |
| Crocosphaera_watsonii_WH_0003 | 5.6 | 37.7 | 5611 | 2 | 1 | 0 | 0 | N | I | marine |
| Crocosphaera_watsonii_WH_0005 | 6.0 | 37.7 | 4681 | 2 | 1 | 0 | 0 | N | I | marine |
| Crocosphaera_watsonii_WH_0401 | 4.5 | 37.7 | 3645 | 2 | 1 | 0 | 0 | N | I | marine |
| Crocosphaera_watsonii_WH_0402 | 5.9 | 37.7 | 4374 | 2 | 1 | 0 | 0 | N | I | marine |
| Crocosphaera_watsonii_WH_8501 | 6.2 | 37.1 | 7191 | 2 | 1 | 0 | 0 | N | I | marine |
| Crocosphaera_watsonii_WH_8502 | 4.7 | 37.6 | 3846 | 2 | 1 | 0 | 0 | N | I | marine |
| Cyanothece_sp._ATCC_51142 | 5.5 | 37.9 | 5159 | 92 | 18 | 1 | 0.06 | B | I | marine |
| Cyanothece_sp._ATCC_51472 | 5.4 | 39.8 | 5216 | 266 | 85 | 15 | 0.18 | B | I | marine |
| Cyanothece_sp._PCC_7425 | 5.8 | 50.6 | 5440 | 266 | 85 | 15 | 0.18 | N | I | freshwater |
| Cyanothece_sp._PCC_7822 | 7.8 | 40.1 | 7382 | 266 | 85 | 15 | 0.18 | N | I | freshwater |
| Cyanothece_sp._PCC_8801 | 4.8 | 39.8 | 4651 | 266 | 85 | 15 | 0.18 | N | I | freshwater |
| Cyanothece_sp._PCC_8802 | 4.8 | 39.8 | 4694 | 266 | 85 | 15 | 0.18 | N | I | freshwater |
| Cylindrospermopsis_raciborskii_CS-505 | 3.9 | 40.2 | 3778 | 865 | 759 | 365 | 0.48 | B | IV | freshwater |
| Cylindrospermopsis_raciborskii_UNSW_506 | 4.2 | 42.0 | 6445 | 865 | 759 | 365 | 0.48 | B | IV | freshwater |
| Dactylococcopsis_salina_PCC_8305 | 3.8 | 42.4 | 4168 | 13 | 4 | 0 | 0 | N | I | freshwater |
| Dolichospermum_circinale_AWQC310F | 4.4 | 37.5 | 4321 | 9 | 9 | 4 | 0.44 | B | IV | freshwater |
| Geminocystis_herdmanii_PCC_6308 | 8.5 | 34.3 | 8600 | 5 | 5 | 0 | 0 | N | I | freshwater |
| Halothece_sp._PCC_7418 | 4.2 | 42.9 | 4076 | 24 | 7 | 0 | 0 | N | I | freshwater |
| Leptolyngbya_boryana_PCC_6306 | 7.3 | 47.0 | 7442 | 31 | 5 | 0 | 0 | N | III | freshwater |
| Leptolyngbya_sp._2LT21S03 | 7.8 | 51.4 | 8707 | 361 | 148 | 16 | 0.11 | N | III | marine |
| Leptolyngbya_sp._PCC_6406 | 5.6 | 43.9 | 5350 | 361 | 148 | 16 | 0.11 | N | III | freshwater |
| Lyngbya_majuscula_3L | 8.5 | 43.7 | 6085 | 979 | 230 | 55 | 0.24 | B | III | marine |
| Lyngbya_sp._PCC_8106 | 7.0 | 41.1 | 6145 | 399 | 149 | 15 | 0.10 | N | III | marine |
| Microcystis_aeruginosa_DIANCHI905 | 4.8 | 42.6 | 5174 | 5011 | 3751 | 2155 | 0.57 | B | I | freshwater |
| Microcystis_aeruginosa_NIES-843 | 5.8 | 42.3 | 5777 | 5011 | 3751 | 2155 | 0.57 | B | I | freshwater |
| Microcystis_aeruginosa_PCC_7941 | 4.7 | 43.0 | 4973 | 5011 | 3751 | 2155 | 0.57 | B | I | freshwater |
| Microcystis_aeruginosa_PCC_9432 | 4.9 | 43.0 | 5191 | 5011 | 3751 | 2155 | 0.57 | B | I | freshwater |
| Microcystis_aeruginosa_PCC_9443 | 5.0 | 43.0 | 5447 | 5011 | 3751 | 2155 | 0.57 | B | I | freshwater |
| Microcystis_aeruginosa_PCC_9701 | 4.6 | 43.0 | 4955 | 5011 | 3751 | 2155 | 0.57 | B | I | freshwater |
| Microcystis_aeruginosa_PCC_9717 | 5.1 | 43.0 | 5737 | 5011 | 3751 | 2155 | 0.57 | B | I | freshwater |
| Microcystis_aeruginosa_PCC_9806 | 4.2 | 43.0 | 4538 | 5011 | 3751 | 2155 | 0.57 | B | I | freshwater |
| Microcystis_aeruginosa_PCC_9807 | 5.0 | 43.0 | 5456 | 5011 | 3751 | 2155 | 0.57 | B | I | freshwater |
| Microcystis_aeruginosa_PCC_9808 | 5.0 | 43.0 | 5306 | 5011 | 3751 | 2155 | 0.57 | B | I | freshwater |
| Microcystis_aeruginosa_PCC_9809 | 4.9 | 43.0 | 5356 | 5011 | 3751 | 2155 | 0.57 | B | I | freshwater |
| Microcystis_aeruginosa_TAIHU98 | 4.8 | 42.0 | 5313 | 5011 | 3751 | 2155 | 0.57 | B | I | freshwater |
| Microcystis_sp._T1-4 | 4.6 | 43.0 | 5003 | 937 | 689 | 334 | 0.48 | B | I | freshwater |
| Myxosarcina_sp._GI1 | 7.1 | 40.1 | 6871 | 13 | 3 | 0 | 0 | N | II | marine |
| Nodularia_spumigena_CCY9414 | 5.3 | 41.3 | 5014 | 480 | 402 | 253 | 0.63 | B | IV | marine |
| Nostoc_sp._PCC_7107 | 6.3 | 40.0 | 5928 | 1141 | 228 | 26 | 0.11 | B | IV | freshwater |
| Nostoc_sp._PCC_7120 | 7.2 | 41.3 | 6694 | 1141 | 228 | 26 | 0.11 | B | IV | freshwater |
| Nostoc_sp._PCC_7524 | 6.7 | 41.2 | 5451 | 1141 | 228 | 26 | 0.11 | B | IV | freshwater |
| Oscillatoria_formosa_PCC_6407 | 6.9 | 43.4 | 6736 | 23 | 15 | 2 | 0.13 | B | III | freshwater |
| Oscillatoria_nigro-viridis_PCC_7112 | 8.0 | 46.0 | 8289 | 3 | 1 | 1 | 1.00 | B | III | freshwater |
| Planktothrix_agardhii_NIVA-CYA_126/8 | 5.5 | 39.5 | 4489 | 398 | 356 | 200 | 0.56 | B | III | freshwater |
| Planktothrix_agardhii_NIVA-CYA_15 | 5.3 | 39.5 | 5020 | 398 | 356 | 200 | 0.56 | B | III | freshwater |
| Planktothrix_agardhii_NIVA-CYA_34 | 5.5 | 39.5 | 5131 | 398 | 356 | 200 | 0.56 | B | III | freshwater |
| Planktothrix_agardhii_NIVA-CYA_56/3 | 5.5 | 39.5 | 5162 | 398 | 356 | 200 | 0.56 | B | III | freshwater |
| Planktothrix_mougeotii_NIVA-CYA_405 | 5.4 | 39.5 | 5174 | 398 | 356 | 200 | 0.56 | B | III | freshwater |
| Planktothrix_prolifica_NIVA-CYA_406 | 5.6 | 39.5 | 5361 | 398 | 356 | 200 | 0.56 | B | III | freshwater |
| Planktothrix_prolifica_NIVA-CYA_540 | 5.5 | 39.5 | 5181 | 398 | 356 | 200 | 0.56 | B | III | freshwater |
| Planktothrix_rubescens_NIVA-CYA_407 | 5.4 | 39.5 | 5094 | 398 | 356 | 200 | 0.56 | B | III | freshwater |
| Planktothrix_rubescens_NIVA-CYA_98 | 5.6 | 39.5 | 5285 | 398 | 356 | 200 | 0.56 | B | III | freshwater |
| Pleurocapsa_sp._PCC_7319 | 7.4 | 38.7 | 6991 | 15 | 6 | 0 | 0 | N | II | freshwater |
| Prochlorococcus_marinus_bv._HNLC1 | 1.7 | 31.2 | 1961 | 184 | 100 | 0 | 0 | N | I | marine |
| Prochlorococcus_marinus_str._EQPAC1 | 1.7 | 30.8 | 1958 | 184 | 100 | 0 | 0 | N | I | marine |
| Prochlorococcus_marinus_str._GP2 | 1.6 | 31.2 | 1880 | 184 | 100 | 0 | 0 | N | I | marine |
| Prochlorococcus_marinus_str._LG | 1.8 | 36.4 | 1983 | 184 | 100 | 0 | 0 | N | I | marine |
| Prochlorococcus_marinus_str._MIT_9107 | 1.7 | 31.0 | 1994 | 184 | 100 | 0 | 0 | N | I | marine |
| Prochlorococcus_marinus_str._MIT_9116 | 1.7 | 31.0 | 1984 | 184 | 100 | 0 | 0 | N | I | marine |
| Prochlorococcus_marinus_str._MIT_9123 | 1.7 | 31.0 | 1999 | 184 | 100 | 0 | 0 | N | I | marine |
| Prochlorococcus_marinus_str._MIT_9201 | 1.7 | 31.3 | 1986 | 184 | 100 | 0 | 0 | N | I | marine |
| Prochlorococcus_marinus_str._MIT_9215 | 1.7 | 31.1 | 2047 | 184 | 100 | 0 | 0 | N | I | marine |
| Prochlorococcus_marinus_str._MIT_9302 | 1.7 | 31.1 | 2016 | 184 | 100 | 0 | 0 | N | I | marine |
| Prochlorococcus_marinus_str._MIT_9311 | 1.7 | 31.2 | 1979 | 184 | 100 | 0 | 0 | N | I | marine |
| Prochlorococcus_marinus_str._MIT_9313 | 2.4 | 50.7 | 2873 | 184 | 100 | 0 | 0 | N | I | marine |
| Prochlorococcus_marinus_str._MIT_9314 | 1.7 | 31.2 | 1979 | 184 | 100 | 0 | 0 | N | I | marine |
| Prochlorococcus_marinus_str._MIT_9321 | 1.7 | 31.2 | 1962 | 184 | 100 | 0 | 0 | N | I | marine |
| Prochlorococcus_marinus_str._MIT_9322 | 1.7 | 31.2 | 1961 | 184 | 100 | 0 | 0 | N | I | marine |
| Prochlorococcus_marinus_str._MIT_9401 | 1.7 | 31.2 | 1969 | 184 | 100 | 0 | 0 | N | I | marine |
| Prochlorococcus_marinus_str._PAC1 | 1.8 | 35.1 | 2254 | 184 | 100 | 0 | 0 | N | I | marine |
| Prochlorococcus_marinus_str._SB | 1.7 | 31.5 | 1935 | 184 | 100 | 0 | 0 | N | I | marine |
| Prochlorococcus_marinus_str._SS2 | 1.8 | 36.4 | 1987 | 184 | 100 | 0 | 0 | N | I | marine |
| Prochlorococcus_marinus_str._SS35 | 1.8 | 36.4 | 1988 | 184 | 100 | 0 | 0 | N | I | marine |
| Prochlorococcus_marinus_str._SS51 | 1.7 | 36.4 | 1976 | 184 | 100 | 0 | 0 | N | I | marine |
| Prochlorococcus_sp._MIT_0601 | 1.7 | 37.0 | 1935 | 281 | 195 | 0 | 0 | N | I | marine |
| Prochlorococcus_sp._MIT_0602 | 1.8 | 36.3 | 1997 | 281 | 195 | 0 | 0 | N | I | marine |
| Prochlorococcus_sp._MIT_0603 | 1.8 | 36.4 | 2004 | 281 | 195 | 0 | 0 | N | I | marine |
| Prochlorococcus_sp._MIT_0604 | 1.8 | 31.2 | 2092 | 281 | 195 | 0 | 0 | N | I | marine |
| Prochlorococcus_sp._MIT_0701 | 2.6 | 50.6 | 3082 | 281 | 195 | 0 | 0 | N | I | marine |
| Prochlorococcus_sp._MIT_0702 | 2.6 | 50.6 | 3084 | 281 | 195 | 0 | 0 | N | I | marine |
| Prochlorococcus_sp._MIT_0703 | 2.6 | 50.6 | 3078 | 281 | 195 | 0 | 0 | N | I | marine |
| Prochlorococcus_sp._MIT_0801 | 1.9 | 34.9 | 2278 | 281 | 195 | 0 | 0 | N | I | marine |
| Prochlorococcus_sp._SS52 | 1.8 | 36.4 | 1987 | 281 | 195 | 0 | 0 | N | I | marine |
| Prochlorothrix_hollandica_PCC_9006 | 5.6 | 54.4 | 5510 | 123 | 27 | 0 | 0 | N | III | freshwater |
| Raphidiopsis_brookii_D9 | 3.2 | 40.1 | 3234 | 15 | 13 | 7 | 0.54 | B | III | freshwater |
| Rivularia_sp._PCC_7116 | 8.7 | 38.0 | 7389 | 28 | 17 | 0 | 0 | N | IV | marine |
| Rubidibacter_lacunae_KORDI_51-2 | 4.1 | 56.2 | 4254 | 2 | 2 | 0 | 0 | N | I | freshwater |
| Spirulina_major_PCC_6313 | 5.1 | 53.0 | 4408 | 278 | 93 | 0 | 0 | N | III | freshwater |
| Spirulina_subsalsa_PCC_9445 | 5.3 | 47.0 | 5180 | 50 | 24 | 0 | 0 | N | III | freshwater |
| Stanieria_cyanosphaera_PCC_7437 | 5.5 | 36.0 | 5163 |  | 0 | 0 | 0 | N | I | freshwater |
| Synechococcus_elongatus_PCC_7942 | 2.7 | 55.4 | 2915 | 1378 | 260 | 3 | 0.01 | N | I | marine |
| Synechococcus_sp._CC9616 | 2.6 | 56.5 | 2964 | 3024 | 838 | 30 | 0.04 | N | I | marine |
| Synechococcus_sp._CC9902 | 2.2 | 54.2 | 2561 | 3024 | 838 | 30 | 0.04 | N | I | marine |
| Synechococcus_sp._KORDI-100 | 2.8 | 57.5 | 3121 | 3024 | 838 | 30 | 0.04 | N | I | marine |
| Synechococcus_sp._KORDI-49 | 2.6 | 61.4 | 2660 | 3024 | 838 | 30 | 0.04 | N | I | marine |
| Synechococcus_sp._KORDI-52 | 2.6 | 59.1 | 2921 | 3024 | 838 | 30 | 0.04 | N | I | marine |
| Synechococcus_sp._WH_8016 | 2.7 | 54.0 | 3174 | 3024 | 838 | 30 | 0.04 | N | I | marine |
| Synechococcus_sp._WH_8102 | 2.4 | 59.4 | 2690 | 3024 | 838 | 30 | 0.04 | N | I | marine |
| Synechocystis_sp._PCC_6803 | 3.9 | 47.4 | 3917 | 3024 | 838 | 30 | 0.04 | N | I | freshwater |
| Trichodesmium_erythraeum_21-75 | 7.0 | 33.9 | 5878 | 212 | 155 | 71 | 0.46 | B | III | marine |
| Trichodesmium_erythraeum_IMS101 | 7.8 | 34.1 | 6500 | 212 | 155 | 71 | 0.46 | B | III | marine |
| Xenococcus_sp._PCC_7305 | 5.9 | 40.0 | 5510 | 11 | 4 | 0 | 0 | N | I | marine |

Table S2. The classification of central and query pathways and their protein reference sources.

|  |  | Category | Source | Literature |
| --- | --- | --- | --- | --- |
| Central Metabolism |  |  | SEED |  |
|  | Glycolysis and gluconeogenesis | *Carbon* | SEED |  |
|  | Entner–Doudoroff pathway | *Carbon* | SEED |  |
|  | Pentose phosphate pathway | *Carbon* | SEED |  |
|  | Tricarboxylic acid cycle | *Carbon* | SEED |  |
|  | Glyoxylate cycle | *Carbon* | SEED |  |
|  | Phycobilisome | *Carbon* | SEED |  |
|  | CO2 concentrating mechanism | *Carbon* | SEED  KEGG |  |
|  | photosystem II | *Carbon* | SEED  KEGG |  |
|  | photosystem I | *Carbon* | SEED  KEGG |  |
|  | Calvin cycle | *Carbon* | SEED  KEGG |  |
|  | respiratory and photosynthetic electron transport chain | *Carbon* | SEED  KEGG |  |
| Accessory metabolism | Nutrient assimilation | *Nitrogen* |  |  |
|  |  | Ammonia | CB |  |
|  |  | Nitrate/Nitrite | CB |  |
|  |  | N_2_ fixation | CB |  |
|  |  | Urea | CB |  |
|  |  | *Phosphorus* |  |  |
|  |  | Phosphate | HB |  |
|  |  | Glycerol-3-phosphate | HB |  |
|  |  | Glycerophosphoryl diesters | HB |  |
|  |  | Phosphonate | HB |  |
|  |  | *Sulfur* |  |  |
|  |  | Sulfate/Thiosulfate | HB |  |
|  |  | Sulfonate | HB |  |
|  |  | *Organics* |  |  |
|  |  | Carbohydrate | HB |  |
|  |  | *N*-acetyl glucosamine | HB |  |
|  |  | Amino acids | HB |  |
|  |  | *Trace metal* |  |  |
|  |  | MnO_4_^2-^ | HB |  |
|  |  | Cu^2+^ | HB |  |
|  |  | Mn^2+^ | HB |  |
|  |  | Mg^2+^ | HB |  |
|  |  | Fe^3+^ | HB/CB |  |
|  |  | Ni^2+^ | HB |  |
|  |  | *Vitamin* |  |  |
|  |  | Thiamine | HB |  |
|  |  | Riboflavin | HB |  |
|  |  | Niacin | HB |  |
|  |  | Biotin | HB |  |
|  |  | Folate | HB |  |
|  |  | Cobalamin | HB |  |
|  | Stress resistance | *Heavy metal* |  |  |
|  |  | Cu^+^ | HB |  |
|  |  | CrO_4_^2+^ | HB |  |
|  |  | Ni^2+^ | HB |  |
|  |  | Zn^2+^ | HB |  |
|  |  | Zn^2+^/ Co^2+^/Cd^2+^ | HB |  |
|  |  | *Multidrug* | HB |  |
|  |  | *Antibiotics/Inhibitors* | HB |  |
|  |  | *UV radiation* | CB |  |
|  |  | *Toxin production* | CB |  |
|  |  | *Low temperature* |  |  |
|  |  | (Poly-) unsaturated fatty acids | HB/CB |  |
|  |  | *Buoyancy regulation* | CB |  |
|  |  | *Osmoprotectant* | HB/CB |  |
|  |  | *Redox balance* | HB/CB |  |

^a^CB: cyanobacteria; HB: heterotrophic bacteria.
