# Supplementary material for: The Trait Repertoire Enabling Cyanobacteria to Bloom Assessed through Comparative Genomic Complexity and Metatranscriptomics
Source: mBio. 2020 Jun 30;11(3):e01155-20. doi: 10.1128/mBio.01155-20 (PMC7327172; doi:10.1128/mBio.01155-20)
Supplement: TEXT S1 [file mBio.01155-20-s0001.docx]

# Supplementary Methodology

# *The trait repertoire enabling cyanobacteria to bloom assessed through comparative genomic complexity and metatranscriptomics*

Huansheng Cao^1,2^, Yohei Shimura^3^, Morgan M. Steffen^4^, Zhou Yang^5^, Jingrang Lu^6^, Allen Joel^6^, Landon Jenkins^2^, Masanobu Kawachi^3^, and Yanbin Yin^1,7*^, Ferran Garcia-Pichel^2*^

^1^Department of Biological Sciences, Northern Illinois University, DeKalb, IL 60115, USA ^2^Biodesign Center for Fundamental and Applied Microbiomics, Arizona State University, Tempe, AZ 85287, USA

^3^National Institute for Environmental Studies, Tsukuba, Ibaraki 305-8506, Japan

^4^Biology Department, James Madison University, Harrisonburg, Harrisonburg, VA 22807,

^5^Jiangsu Key Laboratory for Biodiversity and Biotechnology, School of Biological Sciences, Nanjing Normal University, Nanjing, Jiangsu, China

^6^U.S. Environmental Protection Agency Office of Research and Development, Cincinnati, OH, USA

^7^Nebraska Food for Health Center, Department of Food Science and Technology, University of Nebraska - Lincoln, Lincoln, NE 68588, USA

^*^Corresponding authors (ferran@asu.edu; yyin@unl.edu).

# Materials and Methods

## References for methods and for Tables S1 and S2

# Materials and Methods

## Phylogeny construction

The 16S sequences were extracted from each genome and aligned using MAFFT version 6.2 ([1](#_ENREF_1)) with default parameters and then a approximately maximum-likelihood phylogenetic tree was built using FastTree 2 ([2](#_ENREF_2)); the tree was visualized using iTOL v3 ([3](#_ENREF_3)).

## Metatranscriptomic analyses

We collected CyanoHAB water samples to measure metatranscriptomes of CyanoHABs from eutrophic Harsha Lake and Lake Erie, respectively. Harsha Lake is an eutrophic Lake with annual *M. aeruginosa* blooms ([4](#_ENREF_4)). Surface water (100–200 mL) during blooms at the Harsha Buoy site (39.032 N, 84.137 W) was collected in duplicate on June 20, 24, and 29, 2016, and water samples at east fork lake at drinking water treatment plant intake (EFLS; 39.037 N, 84.138 W) were collected on June 25 and 30, 2016 onto Durapore polyvinylidene fluoride (PVDF) filters (0.45 μm, MilliPore, Foster City, CA). RNA extraction and sequencing were performed as described ([4](#_ENREF_4)). The raw reads were deposited in NCBI SRA (accession number SRP212026). The samples from Lake Erie were collected during peak blooms at three sites on the same day as described ([5](#_ENREF_5)) and deposited into NCBI SRA (SRP056145). We filtered all the raw reads with a quality cutoff of 26 and trimmed low-quality ends with FASTX (http://hannonlab.cshl.edu/fastx_toolkit/). Clean reads were mapped to the *M.* *aeruginosa* NIES-843 genome (NC_010296.1) with CLC Genomics Workbench 12 (https://www.qiagenbioinformatics.com) (Qiagen, CA, USA) and the gene expression levels were expressed as the normalized TPM (Transcripts Per Million) to correct the differences in the number of mapped reads and gene length ([6](#_ENREF_6)).

Those in eutrophic waters included our own datasets above during CyanoHABs, which were all mapped to the *M.* *aeruginosa* NIES-843 genome. The metatranscriptomes of oligotrophic waters included those from Sparkling Lake (oligotrohic freshwater) (PRJNA368303, PRJNA368305 and PRJNA368306) ([7](#_ENREF_7)) and the Arctic Ocean (oligotrophic ocean) (PRJNA520208, PRJNA520210 and PRJNA520220), which were also analyzed as detailed above but mapped to reference genomes close to their own dominant cyanobacterial species, *Oscillatoria nigro-viridis* PCC 7112 and *Synechococcus* sp WH 8102.

**References**

1. K. Katoh, K.-i. Kuma, H. Toh, T. Miyata, MAFFT version 5: improvement in accuracy of multiple sequence alignment. *Nucl. Acids Res.* **33**, 511-518 (2005).

2. M. N. Price, P. S. Dehal, A. P. Arkin, FastTree 2 – Approximately maximum-likelihood trees for large alignments. *PLOS ONE* **5**, e9490 (2010).

3. I. Letunic, P. Bork, Interactive tree of life (iTOL) v3: an online tool for the display and annotation of phylogenetic and other trees. *Nucl. Acids Res.* **44**, W242-W245 (2016).

4. J. Lu, B. Zhu, I. Struewing, N. Xu, S. Duan, Nitrogen–phosphorus-associated metabolic activities during the development of a cyanobacterial bloom revealed by metatranscriptomics. *Sci. Rep.* **9**, 2480 (2019).

5. M. M. Steffen *et al.*, Metatranscriptomic evidence for co-occurring top-down and bottom-up controls on toxic cyanobacterial communities. *Applied and Environmental Microbiology* **81**, 3268-3276 (2015).

6. B. Li, C. N. Dewey, RSEM: accurate transcript quantification from RNA-Seq data with or without a reference genome. *BMC Bioinformatics* **12**, 323 (2011).

7. D. C. Richardson, C. C. Carey, D. A. Bruesewitz, K. C. Weathers, Intra- and inter-annual variability in metabolism in an oligotrophic lake. *Aquat. Sci.* **79**, 319-333 (2017).

8. R. Overbeek *et al.*, The subsystems approach to genome annotation and its use in the project to annotate 1000 genomes. *Nucl. Acids Res.* **33**, 5691-5702 (2005).

9. M. Kanehisa *et al.*, Data, information, knowledge and principle: back to metabolism in KEGG. *Nucl. Acids Res.* **42**, D199-D205 (2014).

10. M. I. Muro-Pastor, J. Reyes, F. Florencio, Ammonium assimilation in cyanobacteria. *Photosynth Res* **83**, 135-150 (2005).

11. Y. Ohashi *et al.*, Regulation of nitrate assimilation in cyanobacteria. *Journal of Experimental Botany* **62**, 1411-1424 (2011).

12. J. Espinosa, K. Forchhammer, A. Contreras, Role of the *Synechococcus* PCC 7942 nitrogen regulator protein PipX in NtcA-controlled processes. *Microbiology* **153**, 711-718 (2007).

13. K. Kumar, R. A. Mella-Herrera, J. W. Golden, Cyanobacterial heterocysts. *Cold Spring Harb. Perspect. Biol.* **2** (2010).

14. A. Valladares, M. L. Montesinos, A. Herrero, E. Flores, An ABC-type, high-affinity urea permease identified in cyanobacteria. *Mol. Microbiol.* **43**, 703-715 (2002).

15. M. G. Lamarche, B. L. Wanner, S. Crépin, J. Harel, The phosphate regulon and bacterial virulence: a regulatory network connecting phosphate homeostasis and pathogenesis. *FEMS Microbiol. Rev.* **32**, 461-473 (2008).

16. B. L. Wanner, "Phosphorous assimilation and control of the phosphate regulon" in Escherichia coli and Salmonella: Cellular and Molecular Biology*,* F. C. Neidhardt, Ed. (ASM, Washington D.C., 1996), vol. 1, chap. 8, pp. 1357-1381.

17. M. A. Kertesz, Bacterial transporters for sulfate and organosulfur compounds. *Res. Microbiol.* **152**, 279-290 (2001).

18. E. Aguilar-Barajas, C. Díaz-Pérez, M. Ramírez-Díaz, H. Riveros-Rosas, C. Cervantes, Bacterial transport of sulfate, molybdate, and related oxyanions. *Biometals* **24**, 687-707 (2011).

19. J. van der Ploeg, E. Eichhorn, T. Leisinger, Sulfonate-sulfur metabolism and its regulation in *Escherichia coli*. *Arch. Microbiol.* **176**, 1-8 (2001).

20. T. Nakatani *et al.*, Enhancement of thioredoxin/glutaredoxin-mediated L-cysteine synthesis from S-sulfocysteine increases L-cysteine production in *Escherichia coli*. *Microbial Cell Factories* **11**, 62 (2012).

21. L. F. Wu, M. A. Mandrand-Berthelot, A family of homologous substrate-binding proteins with a broad range of substrate specificity and dissimilar biological functions. *Biochimie* **77**, 744-750 (1995).

22. Y.-F. Wang, R. Dutzler, P. J. Rizkallah, J. P. Rosenbusch, T. Schirmer, Channel specificity: structural basis for sugar discrimination and differential flux rates in maltoporin. *J. Mol. Biol.* **272**, 56-63 (1997).

23. J. Nedoma, J. Vrba, J. Hejzlar, K. Simek, V. Straskrabova, *N*-acetylglucosamine dynamics in freshwater environments: Concentration of amino sugars, extracellular enzyme activities, and microbial uptake. *Limnol. Oceanogr.* **39**, 1088-1100 (1994).

24. C. Yang *et al.*, Comparative genomics and experimental characterization of N-acetylglucosamine utilization pathway of *Shewanella oneidensis*. *J. Biol. Chem.* **281**, 29872-29885 (2006).

25. J. Saier, Milton H., Families of transmembrane transporters selective for amino acids and their derivatives. *Microbiology* **146**, 1775-1795 (2000).

26. A. H. F. Hosie, P. S. Poole, Bacterial ABC transporters of amino acids. *Res. Microbiol.* **152**, 259-270 (2001).

27. D. L. Walshaw, P. S. Poole, The general l-amino acid permease of *Rhizobium leguminosarum* is an ABC uptake system that also influences efflux of solutes. *Mol. Microbiol.* **21**, 1239-1252 (1996).

28. M. D. Adams *et al.*, Nucleotide sequence and genetic characterization reveal six essential genes for the LIV-I and LS transport systems of *Escherichia coli*. *J. Biol. Chem.* **265**, 11436-11443 (1990).

29. M. Rahmanian, D. R. Claus, D. L. Oxender, Multiplicity of leucine transport systems in *Escherichia coli* K-12. *J. Bacteriol.* **116**, 1258-1266 (1973).

30. V. Bartsevich, H. Pakrasi, Molecular identification of an ABC transporter complex for manganese: analysis of a cyanobacterial mutant strain impaired in the photosynthetic oxygen evolution process. *The EMBO Journal* **14**, 1845-1853 (1995).

31. T. Ogawa *et al.*, A two-component signal transduction pathway regulates manganese homeostasis in *Synechocystis* 6803, a photosynthetic organism. *J. Biol. Chem.* **277**, 28981-28986 (2002).

32. C. Rademacher, B. Masepohl, Copper-responsive gene regulation in bacteria. *Microbiology* **158**, 2451-2464 (2012).

33. M. B. C. Moncrief, M. E. Maguire, Magnesium transport in prokaryotes. *JBIC* **4**, 523-527 (1999).

34. M. Stevanovic, A. Hahn, K. Nicolaisen, O. Mirus, E. Schleiff, The components of the putative iron transport system in the cyanobacterium *Anabaena* sp. PCC 7120. *Environ. Microbiol.* **14**, 1655-1670 (2012).

35. B. Martinac, Y. Saimi, C. Kung, Ion channels in microbes. *Physiol. Rev.* **88**, 1449-1490 (2008).

36. G. C. Gerloff, F. Skoog, Availability of iron and manganese in southern wisconsin lakes for the growth of *Microcystis aeruginosa*. *Ecology* **38**, 552-556 (1957).

37. Y. Zhang, D. Rodionov, M. Gelfand, V. Gladyshev, Comparative genomic analyses of nickel, cobalt and vitamin B12 utilization. *BMC Genomics.* **10**, 78 (2009).

38. E. Webb, K. Claas, D. Downs, thiBPQ encodes an ABC transporter required for transport of thiamine and thiamine pyrophosphate in *Salmonella typhimurium*. *J. Biol. Chem.* **273**, 8946-8950 (1998).

39. C. Vogl *et al.*, Characterization of riboflavin (vitamin B2) transport proteins from *Bacillus subtilis* and *Corynebacterium glutamicum*. *J. Bacteriol.* **189**, 7367-7375 (2007).

40. A. G. Vitreschak, D. A. Rodionov, A. A. Mironov, M. S. Gelfand, Regulation of riboflavin biosynthesis and transport genes in bacteria by transcriptional and translational attenuation. *Nucl. Acids Res.* **30**, 3141-3151 (2002).

41. R. K. Deka, C. A. Brautigam, B. A. Biddy, W. Z. Liu, M. V. Norgard, Evidence for an ABC-type riboflavin transporter system in pathogenic spirochetes. *mBio* **4** (2013).

42. D. A. Rodionov *et al.*, A novel class of modular transporters for vitamins in prokaryotes. *J. Bacteriol.* **191**, 42-51 (2009).

43. A. Eudes *et al.*, Identification of transport-critical residues in a folate transporter from the folate-biopterin transporter (FBT) family. *J. Biol. Chem.* **285**, 2867-2875 (2010).

44. C. Kündig, A. Haimeur, D. Légaré, B. Papadopoulou, M. Ouellette, Increased transport of pteridines compensates for mutations in the high affinity folate transporter and contributes to methotrexate resistance in the protozoan parasite *Leishmania tarentolae*. *The EMBO Journal* **18**, 2342-2351 (1999).

45. L. C. de Veaux, D. S. Clevenson, C. Bradbeer, R. J. Kadner, Identification of the btuCED polypeptides and evidence for their role in vitamin B12 transport in *Escherichia coli*. *J. Bacteriol.* **167**, 920-927 (1986).

46. D. Nies, "Bacterial transition metal Homeostasis" in Molecular Microbiology of Heavy Metals*,* D. Nies, S. Silver, Eds. (Springer Berlin Heidelberg, 2007), vol. 6, chap. 75, pp. 117-142.

47. D. Nies, S. Silver, Ion efflux systems involved in bacterial metal resistances. *J. Ind. Microbiol.* **14**, 186-199 (1995).

48. D. H. Nies, Efflux-mediated heavy metal resistance in prokaryotes. *FEMS Microbiol. Rev.* **27**, 313-339 (2003).

49. H. Nikaido, Multidrug efflux pumps of gram-negative bacteria. *J. Bacteriol.* **178**, 5853-5859 (1996).

50. J. A. Delmar, C.-C. Su, E. W. Yu, Bacterial multidrug efflux transporters. *Annual Review of Biophysics* 10.1146/annurev-biophys-051013-022855 (2013).

51. S. Kumar, M. M. Mukherjee, M. F. Varela, Modulation of bacterial multidrug resistance efflux pumps of the major facilitator superfamily. *International Journal of Bacteriology* **2013**, 15 (2013).

52. E. P. Balskus, C. T. Walsh, The genetic and molecular basis for sunscreen biosynthesis in cyanobacteria. *Science* **329**, 1653-1656 (2010).

53. A. Rantala-Ylinen *et al.*, Anatoxin-a synthetase gene cluster of the cyanobacterium *Anabaena* sp. strain 37 and molecular methods to detect potential producers. *Applied and Environmental Microbiology* **77**, 7271-7278 (2011).

54. T. Mihali, R. Kellmann, B. Neilan, Characterisation of the paralytic shellfish toxin biosynthesis gene clusters in *Anabaena circinalis* AWQC131C and *Aphanizomenon* sp. NH-5. *BMC Biochem.* **10**, 8 (2009).

55. T. K. Mihali, R. Kellmann, J. Muenchhoff, K. D. Barrow, B. A. Neilan, Characterization of the gene cluster responsible for cylindrospermopsin biosynthesis. *Applied and Environmental Microbiology* **74**, 716-722 (2008).

56. M. C. Moffitt, B. A. Neilan, Characterization of the Nodularin Synthetase Gene Cluster and Proposed Theory of the Evolution of Cyanobacterial Hepatotoxins. *Applied and Environmental Microbiology* **70**, 6353-6362 (2004).

57. A. Calteau *et al.*, Phylum-wide comparative genomics unravel the diversity of secondary metabolism in Cyanobacteria. *BMC Genomics.* **15**, 977 (2014).

58. E. Dittmann, D. P. Fewer, B. A. Neilan, Cyanobacterial toxins: biosynthetic routes and evolutionary roots. *FEMS Microbiol. Rev.* **37**, 23-43 (2013).

59. X. Chi *et al.*, Comparative analysis of fatty acid desaturases in cyanobacterial genomes. *Comparative and Functional Genomics* **2008** (2008).

60. H. Okuyama, Y. Orikasa, T. Nishida, K. Watanabe, N. Morita, Bacterial genes responsible for the biosynthesis of eicosapentaenoic and docosahexaenoic acids and their heterologous expression. *Applied and Environmental Microbiology* **73**, 665-670 (2007).

61. H. Takeyama, D. Takeda, K. Yazawa, A. Yamada, T. Matsunaga, Expression of the eicosapentaenoic acid synthesis gene cluster from *Shewanella* sp. in a transgenic marine cyanobacterium, *Synechococcus* sp. *Microbiology* **143**, 2725-2731 (1997).

62. A. Mlouka, K. Comte, A.-M. Castets, C. Bouchier, N. Tandeau de Marsac, The gas vesicle gene cluster from *Microcystis aeruginosa* and DNA rearrangements that lead to loss of cell buoyancy. *J. Bacteriol.* **186**, 2355-2365 (2004).
