# Supplementary material for: The Trait Repertoire Enabling Cyanobacteria to Bloom Assessed through Comparative Genomic Complexity and Metatranscriptomics
Source: mBio. 2020 Jun 30;11(3):e01155-20. doi: 10.1128/mBio.01155-20 (PMC7327172; doi:10.1128/mBio.01155-20)
Supplement: TABLE S2 [file mBio.01155-20-st002.doc]

Table S2. The classification of central and query pathways and their protein reference sources.

|  |  | Category | Source | Literature |
| --- | --- | --- | --- | --- |
| Central Metabolism |  |  | SEED |  |
|  | Glycolysis and gluconeogenesis | *Carbon* | SEED |  |
|  | Entner–Doudoroff pathway | *Carbon* | SEED |  |
|  | Pentose phosphate pathway | *Carbon* | SEED |  |
|  | Tricarboxylic acid cycle | *Carbon* | SEED |  |
|  | Glyoxylate cycle | *Carbon* | SEED |  |
|  | Phycobilisome | *Carbon* | SEED |  |
|  | CO2 concentrating mechanism | *Carbon* | SEED  KEGG |  |
|  | photosystem II | *Carbon* | SEED  KEGG |  |
|  | photosystem I | *Carbon* | SEED  KEGG |  |
|  | Calvin cycle | *Carbon* | SEED  KEGG |  |
|  | respiratory and photosynthetic electron transport chain | *Carbon* | SEED  KEGG |  |
| Accessory metabolism | Nutrient assimilation | *Nitrogen* |  |  |
|  |  | Ammonia | CB |  |
|  |  | Nitrate/Nitrite | CB |  |
|  |  | N_2_ fixation | CB |  |
|  |  | Urea | CB |  |
|  |  | *Phosphorus* |  |  |
|  |  | Phosphate | HB |  |
|  |  | Glycerol-3-phosphate | HB |  |
|  |  | Glycerophosphoryl diesters | HB |  |
|  |  | Phosphonate | HB |  |
|  |  | *Sulfur* |  |  |
|  |  | Sulfate/Thiosulfate | HB |  |
|  |  | Sulfonate | HB |  |
|  |  | *Organics* |  |  |
|  |  | Carbohydrate | HB |  |
|  |  | *N*-acetyl glucosamine | HB |  |
|  |  | Amino acids | HB |  |
|  |  | *Trace metal* |  |  |
|  |  | MnO_4_^2-^ | HB |  |
|  |  | Cu^2+^ | HB |  |
|  |  | Mn^2+^ | HB |  |
|  |  | Mg^2+^ | HB |  |
|  |  | Fe^3+^ | HB/CB |  |
|  |  | Ni^2+^ | HB |  |
|  |  | *Vitamin* |  |  |
|  |  | Thiamine | HB |  |
|  |  | Riboflavin | HB |  |
|  |  | Niacin | HB |  |
|  |  | Biotin | HB |  |
|  |  | Folate | HB |  |
|  |  | Cobalamin | HB |  |
|  | Stress resistance | *Heavy metal* |  |  |
|  |  | Cu^+^ | HB |  |
|  |  | CrO_4_^2+^ | HB |  |
|  |  | Ni^2+^ | HB |  |
|  |  | Zn^2+^ | HB |  |
|  |  | Zn^2+^/ Co^2+^/Cd^2+^ | HB |  |
|  |  | *Multidrug* | HB |  |
|  |  | *Antibiotics/Inhibitors* | HB |  |
|  |  | *UV radiation* | CB |  |
|  |  | *Toxin production* | CB |  |
|  |  | *Low temperature* |  |  |
|  |  | (Poly-) unsaturated fatty acids | HB/CB |  |
|  |  | *Buoyancy regulation* | CB |  |
|  |  | *Osmoprotectant* | HB/CB |  |
|  |  | *Redox balance* | HB/CB |  |

^a^CB: cyanobacteria; HB: heterotrophic bacteria.
