# Supplementary material for: The Trait Repertoire Enabling Cyanobacteria to Bloom Assessed through Comparative Genomic Complexity and Metatranscriptomics
Source: mBio. 2020 Jun 30;11(3):e01155-20. doi: 10.1128/mBio.01155-20 (PMC7327172; doi:10.1128/mBio.01155-20)
Supplement: FIG S4 [file mBio.01155-20-sf004.pdf]

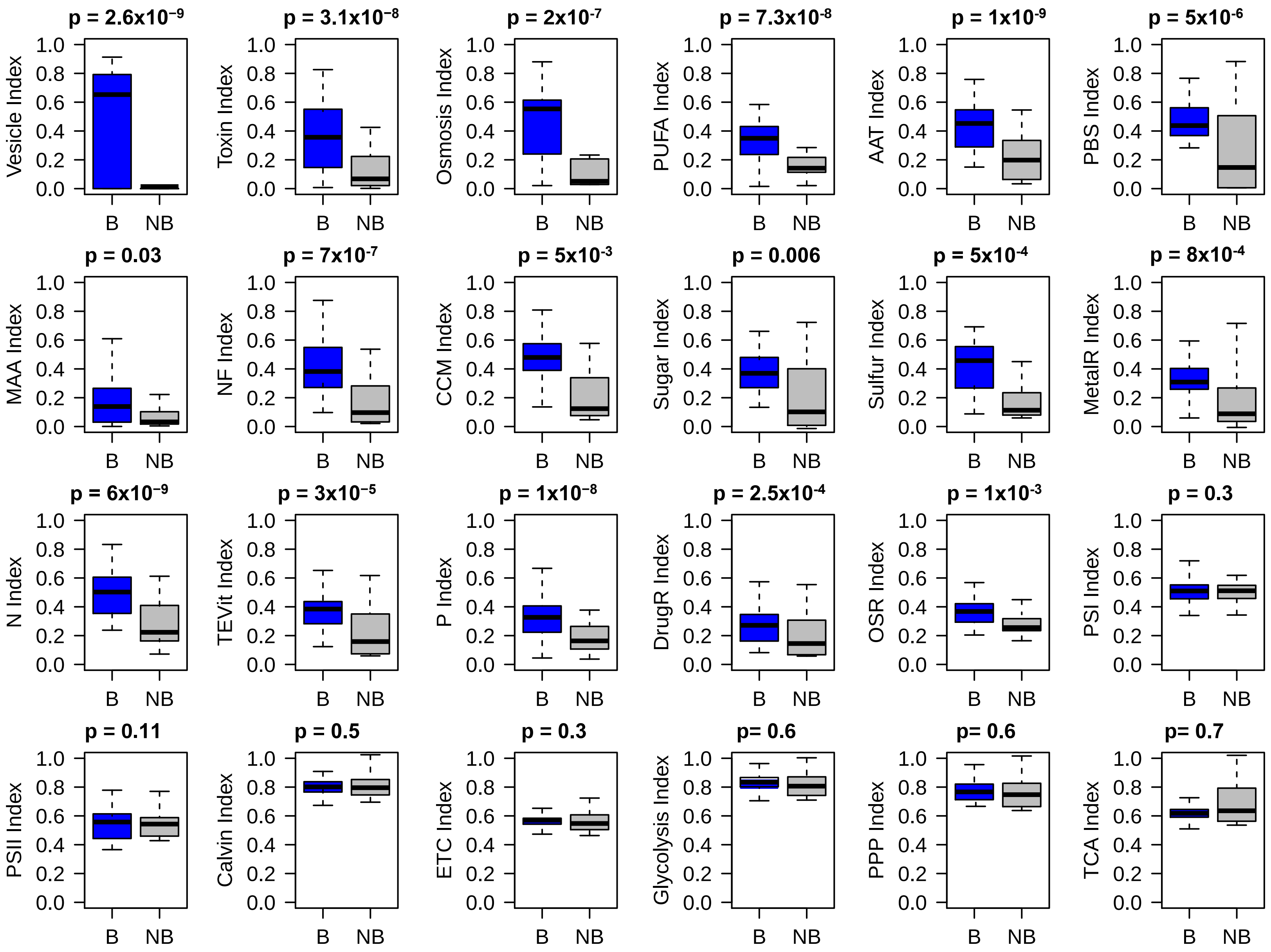

Figure S4. Comparison of the 24 pathways in terms of eight metrics between blooming and non-blooming strains without overrepresented species. B: blooming; nB: non-blooming. *: different from the blooming group at p < 1E-4 levels in Wilcoxon tests
