# Supplementary material for: The Trait Repertoire Enabling Cyanobacteria to Bloom Assessed through Comparative Genomic Complexity and Metatranscriptomics
Source: mBio. 2020 Jun 30;11(3):e01155-20. doi: 10.1128/mBio.01155-20 (PMC7327172; doi:10.1128/mBio.01155-20)
Supplement: FIG S5 [file mBio.01155-20-sf005.pdf]

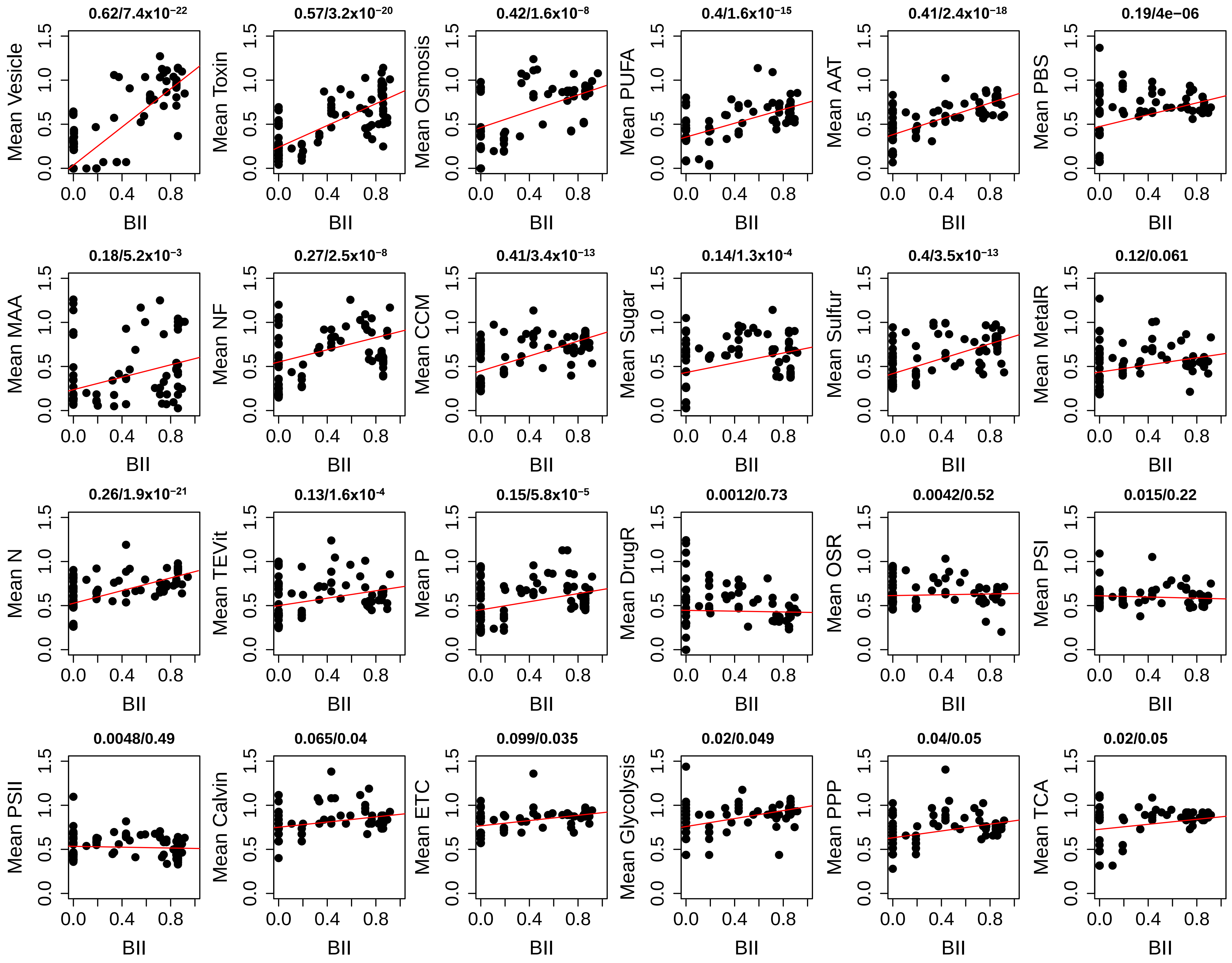

Figure S5. Pearson's correlations between GCI and BII in each pathway. The values on top of each plot represent the coefficient of determination and *p* value, separated by a forward slash.
