# Supplementary material for: The Trait Repertoire Enabling Cyanobacteria to Bloom Assessed through Comparative Genomic Complexity and Metatranscriptomics
Source: mBio. 2020 Jun 30;11(3):e01155-20. doi: 10.1128/mBio.01155-20 (PMC7327172; doi:10.1128/mBio.01155-20)
Supplement: FIG S6 [file mBio.01155-20-sf006.pdf]

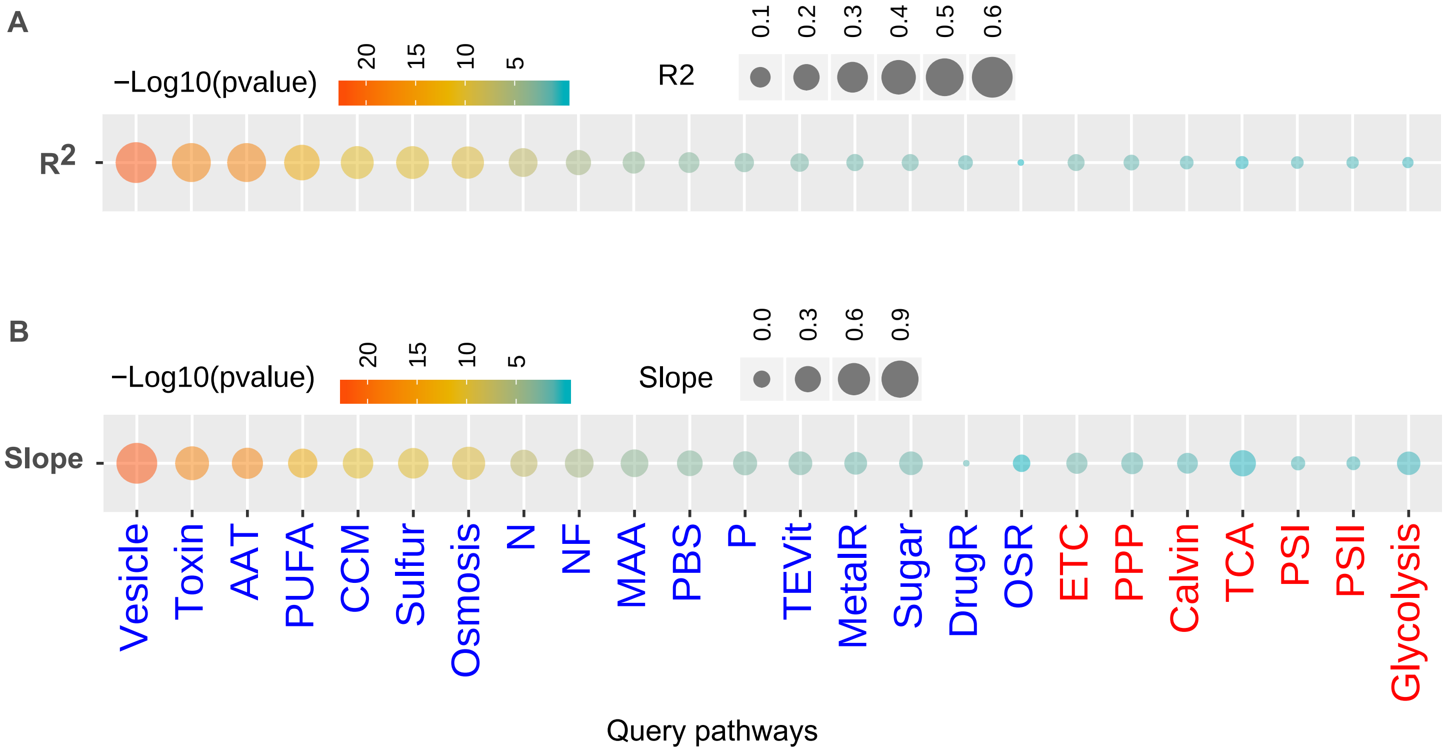

Figure S6. The correlation between the query pathway PCI and BII of all 113 genomes. (A) the coefficients of determination and (B) the slope of the regressions.
