# Supplementary material for: The Trait Repertoire Enabling Cyanobacteria to Bloom Assessed through Comparative Genomic Complexity and Metatranscriptomics
Source: mBio. 2020 Jun 30;11(3):e01155-20. doi: 10.1128/mBio.01155-20 (PMC7327172; doi:10.1128/mBio.01155-20)
Supplement: FIG S3 [file mBio.01155-20-sf003.pdf]

Figure S3. The core and query pathways in this study.

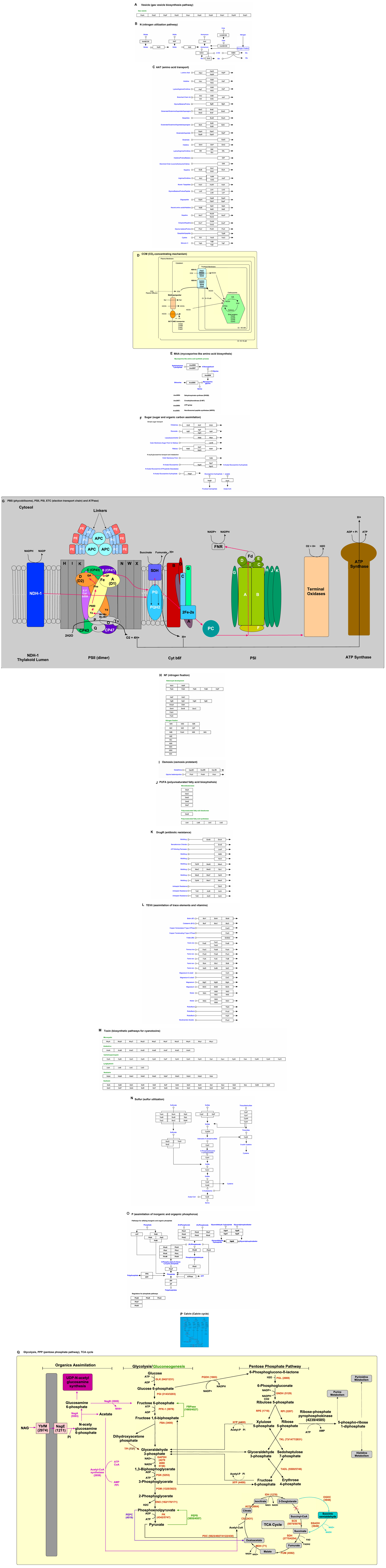

Figure S3. The core and query pathways in this study.
